# Satellite monitoring of bio-fertilizer restoration in olive groves affected by *Xylella fastidiosa* subsp*. pauca*

**DOI:** 10.1038/s41598-023-32170-x

**Published:** 2023-04-07

**Authors:** Palma Blonda, Cristina Tarantino, Marco Scortichini, Sabino Maggi, Maria Tarantino, Maria Adamo

**Affiliations:** 1 Institute of Atmospheric Pollution Research, National Research Council of Italy, c/o Interateneo Physics Department, Via Amendola 173, 70126 Bari, Italy; 2 Research Centre for Olive, Fruit and Citrus Crops, Council for Agricultural Research and Economics, Via di Fioranello 52, 00134 Rome, Italy; 3 Interateneo Physics Department, University of Bari, Via Amendola 173, 70126 Bari, Italy

**Keywords:** Ecology, Environmental sciences

## Abstract

*Xylella fastidiosa* subsp. *pauca* (*Xfp*), has attacked the olive trees in Southern Italy with severe impacts on the olive agro-ecosystem. To reduce both the *Xfp* cell concentration and the disease symptom, a bio-fertilizer restoration technique has been used. Our study applied multi-resolution satellite data to evaluate the effectiveness of such technique at both field and tree scale. For field scale, a time series of High Resolution (HR) Sentinel-2 images, acquired in the months of July and August from 2015 to 2020, was employed. First, four spectral indices from treated and untreated fields were compared. Then, their trends were correlated to meteo-events. For tree-scale, Very High Resolution (VHR) Pléiades images were selected at the closest dates of the Sentinel-2 data to investigate the response to treatments of each different cultivar. All indices from HR and VHR images were higher in treated fields than in those untreated. The analysis of VHR indices revealed that Oliarola Salentina can respond better to treatments than Leccino and Cellina cultivars. All findings were in agreement with in-field PCR results. Hence, HR data could be used to evaluate plant conditions at field level after treatments, while VHR imagery could be used to optimize treatment doses per cultivar.

## Introduction

*Xylella fastidiosa* subsp*. pauca* (*Xfp*) is a quarantine bacterium which can affect olive trees. Among olive groves, this bacterium can create a disease named “olive quick decline syndrome” (OQDS) that incites leaf, twig and branch die-back followed by the final tree collapse^1,2^. The disease is spread through the xylem-sap feeding activity of the adult stage of the *Philaenus spumarius* insect, commonly known as “meadow spittlebug”^3^. Through this insect, the spreading of the disease takes place mainly from the end of spring to early summer^3^. Some wild weeds that latently host the bacterium can act as “reservoir” for the pathogen dispersal^4^. Since the typical *Xfp* lifestyle is restricted to the xylem tissue of plant species, where the bacterium survives and multiplies, field control of the disease has been traditionally retained very difficult^5^. Hence, phytosanitary laws, which are regulated by European and Mediterranean Plant Protection Organization (EPPO), are aimed at reducing the bacterium spreading through tree eradication procedures.

Since October 2013, *Xfp* has affected the olive-agroecosystem found in Salento area of Apulia, Southern Italy. At the time of the detection, Gallipoli and some nearby municipalities were identified as the area of the initial outbreak of OQDS^6^. It must be noted that, at the time of the official record, the disease had already spread over about 8,000–10,000 ha, corresponding to about 1 million olive trees^5^. Such a situation precluded any attempt of pathogen control through tree eradication, in this area.

After one year of the first report, OQDS was detected on about 23,000 ha^7^. Thus, a vast portion of Salento was officially declared “infected”. In order to avoid the further spread of the pathogen, two other areas, adjacent to the infected one, were established as the “containment” and “buffer” zones (Decision of the European Union, 2020).

At present, there are more than 6,500,000 olive trees infected by *Xfp*^8^. According to the most updated European Food Safety Authority (EFSA) report^9^, the overall number of *Xylella* host plant subspecies can range from 423 to 670 species, depending on detection methods applied. Besides *pauca,* in Italy another *Xylella fastidiosa* subspecies, namely, *multiplex* has been found in the Mount Argentario area (Grosseto province, Tuscany) on some wild shrubs of the Mediterranean “macchia”^10^. But so far, the pathogen has not spread further in the nearby areas.

To compute potential future economic impact of the *Xfp* strain in different scenarios, a spatially explicit bio-economic model has recently been developed^11^. The authors of this model report that, the economic potential impact, over 50 years, for Italy, could range from 1.9 billion to 5.2 billion Euros if production would cease after orchards die off (worst-case scenario). If replanting with resistant varieties were feasible, the impact would range from 0.6 billion to 1.6 billion Euros^11^.

In Apulia, to reduce the spread of *Xfp*, besides the eradication of infected trees and all plants found within a 50 m. radius around the infected areas, current policies adopt insect vector control strategies^12^. These include, on the one hand, the reduction of both eggs and insect (*Philaenus spumarius*) juvenile forms through the mechanical removal of the weeds from March to May, depending on fields altitude^4,12^. On the other hand, to limit the insect vector spreading in adult phases, pesticides, i.e., acetamiprid, spinetoram, deltametrina, flupyradifurone, are currently sprayed on olive tree crowns. The number of treatments and in which months to apply are indicated according to in-field *Xfp* monitoring results^12^.

The policies just mentioned are strongly suggested in areas not yet infected, but compulsory to all the entire municipal agro of the containment and buffer areas. At present, such areas include 43 municipalities, for the juvenile phase treatments and 19 municipalities, for the adult phase^12^. In addition, economic incentives are provided to farmers for uprooting old olive trees and investing in new intensive olive groves. Two cultivars, namely Fs-17 and Leccino, have been found tolerant to *Xfp* upon transcriptome profiling^13,14^, thus farmers are encouraged to plant these cultivars. However, due to climate change, intensive agriculture is becoming unsustainable in an area affected by increasing desertification processes (Southern Italy). Every year, the specific areas regulated by the mentioned policies are updated, depending on *Xfp* monitoring results. However, despite massive tree eradication and use of pesticides following the given policies, *Xfp* continues to spread.

Since olive agro-ecosystem constitutes a very unique evergreen woodland covering an area of about 9.000.000 ha^15,16^ in the whole Mediterranean basin, this ecosystem can contribute to climate mitigation and natural resources conservation. Through soil protection, enhanced soil retention and carbon sequestration, olive agro-ecosystem can provide a regulating service. Recent literature has demonstrated the capacity of olive groves to act as a sink of CO2 in the trunk, branches and roots as well as in the underlying soil^17,18^. Fluxes of CO_2_ have been quantified in olive grove fields by using Eddy Covariance Tower (ED) measurements^19^. Such carbon sequestration can be increased when sustainable agricultural practices are implemented^16^. Therefore, the recovery of olive agro-ecosystems is of fundamental importance, not only for climate mitigation and the conservation of the landscape consisting in centenarian and millenarian olive trees^20^, but also for productive/economic reasons^6,21^.

Despite the general opinion that *Xfp* cannot be controlled, a series of studies have been carried out to set up a sustainable control strategy^20,22–24^. A restoration strategy has already been implemented in some experimental fields of Salento, namely in the municipalities of Cannole, facing the Adriatic coast, as well as in Galatone and Nardò, located on the Ionian coast. In the latter municipality, two fields were treated (Nardò A and Nardò B). In these fields, a bio-fertilizer, consisting of the zinc-copper-citric acid known as Dentamet was sprayed on the olive tree crowns, once per month from spring to early autumn since 2016^22^.

Dentamet was chosen after a preliminary evaluation of its bactericidal activity towards *Xylella fastidiosa* and for its significant systematicity within the xylem tissue of olive trees^22^. Effective systematicity within the plant xylem coupled with a bactericidal activity are required for compounds to control the disease and display a satisfactory activity^22^ in open-field conditions. More recently, the same bio-fertilizer has been directly injected into tree trunks by a novel endo-therapeutic precision intravascular injection system^25^. Such internationally-patented bio-fertilizer can provide a relevant systemic activity within the trees and reduce both the disease symptoms and the *Xfp* cell concentration within the local cultivar leaves^23,25^. In addition, upon the treatments, an early reprogramming of some metabolic pathways that re-establish the normal tree physiology has been also found^23^.

The present paper discusses an application of multi-resolution satellite data to evaluate the effectiveness of olive agro-ecosystem restoration action based on spraying the Dentamet bio-fertilizer on tree crowns in the Salento area of Apulia. Although a number of papers have been reported on Earth Observation (EO) monitoring schemes of restoration actions applied to forest ecosystems, at the best of our knowledge, no papers have been presented on EO monitoring of *Xfp* restoration actions. At present, EO techniques have already been used to monitor only the *Xfp* spreading across Salento, but no research has focused on *Xfp* restoration actions. Actually, data from airborne imaging spectroscopy and thermography have been used to detect changes in plant functional traits^26^ induced by *Xfp* spreading. Both Sentinel-2 (S-2) and hyper-spectral data, obtained from airplane campaigns, have also been used^27^. Specifically, S-2 data have been exploited to quantify the extension of areas covered by wilting olive trees, while hyperspectral data have been analysed to extract spectral features to identify early symptoms of OQDS caused by *Xfp*. These techniques have revealed *Xfp* infection before symptoms are visible in olive trees^26^. To validate the presence of the pathogen, all these studies have used in-field samples through the Polymerase Chain Reaction (PCR) technique. The contribution of Solar–Induced chlorophyll Fluorescence (SIF) index and the temperature-based Crop Water Stress Index (CWSI) retrieved from hyperspectral and thermal imageries, respectively, have been analysed through Support Vector Machine algorithms in^28^. The findings have indicated that CWSI can be more useful than SIF for detecting *Xfp* infection when monitoring large areas.

Compared with previous studies focusing on *Xfp* spreading, our paper aims to use multi-resolution satellite and meteo-data to assess the effectiveness of the Dentamet restoration action as well as support the optimization of the bio-fertilizer dosing treatments. To this purpose, first the trends of spectral indices from time series (2015–2020) of freely available HR S-2 data will be analysed at field scale and correlated with meteo-events. The index values of olive trees in treated fields will be compared with the ones in untreated fields over time. Then, to evaluate the response of different cultivars to treatments, VHR Pléiades indices will be analysed at tree scale.

The results obtained from our multi-resolution data will be qualitatively compared with the PCR analysis performed on the same experimental fields^23^. The findings will suggest further Dentamet experimentation on larger areas supported by multiscale EO monitoring.

## Methods

When analysing spectral vegetation index values from satellite images, changes in the indices selected may be related to several causes, including vegetation soil coverage, pruning dates, reduced rain events, heat waves^29^. Thus, pruning dates are reported in Table 1, for each experimental field, to identify possible decreases in the spectral values due to tree biomass reduction after pruning (first subsection). Rain and temperature values were collected in the study period (second subsection). Finally, to minimize the background spectral contribution, only satellite images acquired in July and August were analysed. Actually, during these months, the vegetated background is generally dry in Salento (third subsection).

**Table 1:** Experimental treated (T) and untreated (U) fields with pruning and treatment dates.

Fields	Coordinates	Pruning	Bio-fertilizer treatments					
			2016	2017	2018	2019	2020	2021
**Ionian coast**								
Galatone	T:18.07283; 40.16236	March 2017		July, 11th Aug, 10th	July, 17th Aug, 20th	July, 9th Aug, 8th	July, 16th Aug, 17th	July, 15th Aug, 16th
	U:18.07379; 40.16090	None						
Nardò A	T: 17.98689; 40.19164	April 2019–2021-2022	July, 13th Aug, 17th	July, 7th Aug, 8th	July, 6th Aug, 7th	July, 11th Aug, 12th	July, 10th Aug, 10th	July, 8th Aug, 10th
	U:17.98890; 40.19280	None						
Nardò B	T:17.98879; 40.19108	April 2019–2021-2022	July, 13th Aug, 17th	July, 7th Aug, 8th	July, 6th Aug, 7th	July, 11th Aug, 12th	July, 10th Aug, 10th	July, 9th Aug, 10th
	U:17.98480; 40.18919	None						
**Adriatic coast**								
Cannole	T:18.40285; 40.15128	March 2016–2020-2021	July, 7th Aug, 9th	July, 4th Aug, 3rd	July, 6th Aug, 6th	July, 12th Aug, 12th	July, 14th Aug, 10th	July, 13th Aug, 11th
	U:18.40508; 40.15195	None						

Only July and August treatments reported.

### Experimental fields description and management practices

The olive groves investigated are located in Galatone and Nardò, on the Ionian coast, and Cannole on the Adriatic side. The Nardò area is subdivided into two parts. On the Ionian side olive groves started to show clear signs of *Xfp* in 2014, whereas on the Adriatic coast the infection began in 2016. All fields are characterized by calcareous soil.

The fields consist of trees aging from 40 to 100 years old with local cultivars. Specifically, the Galatone treated field includes three different cultivars, i.e. Leccino, Ogliarola Salentina and Cellina di Nardò, whereas only the latter two cultivars are present both in Cannole and Nardò treated fields. Among these cultivars, Leccino displays both the lowest level of *Xfp* symptoms and the lowest level of bacterial colonization^13,30^. Other cultivars in Salento, i.e., ‘Nocellara Messinese’ and ‘Frantoio’ can display significantly lower symptoms than ‘Ogliarola Salentina’ and ‘Cellina di Nardò’^30^. But the former cultivars are not planted in the experimental fields.

Restoration treatments with Dentamet, at a dose of 3.9 L per ha, were sprayed on olive crowns, once per month, from spring to early autumn (Table 1)^23^. The treatment was trough foliar application. As control, surrounding olive groves that were not subjected to any restoration, with similar soil condition and tree density, were selected to evaluate differences in the vegetation status. The fields analysed are listed in Table 1.

### In-field meteo-data

**Figure 1:**
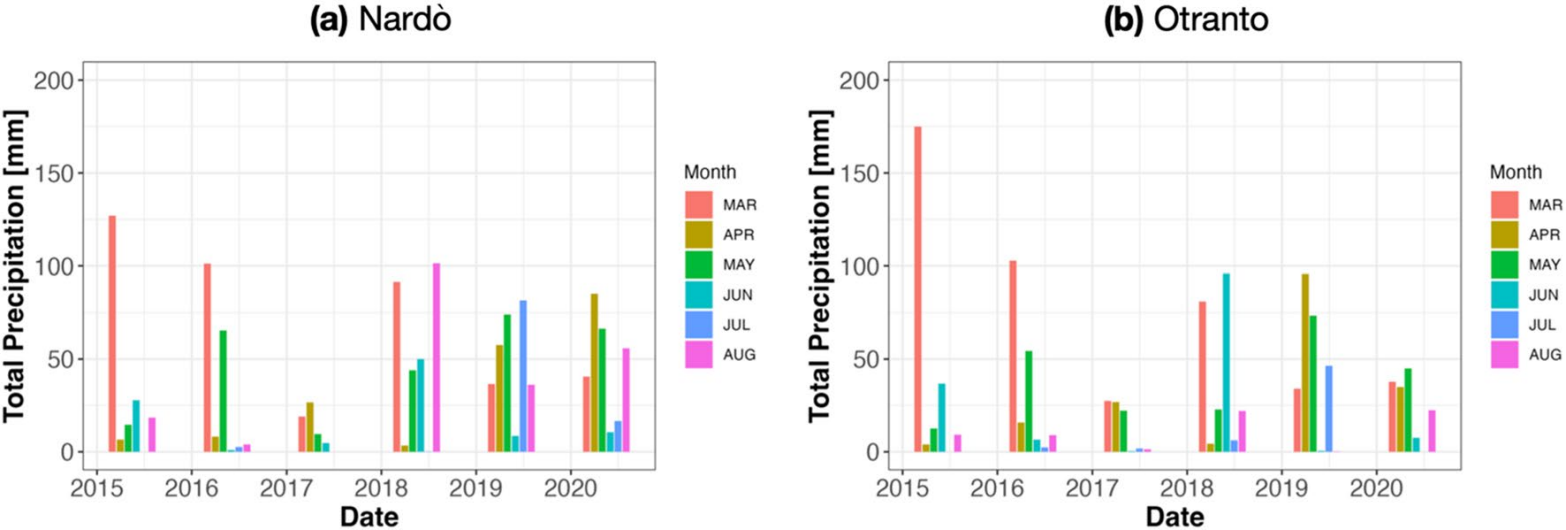
Total monthly precipitation (Meteo-data were collected from the Nardò meteo-station close to the fields Nardò A, Nardò B as well as Galatone, and from the Otranto one which is close to Cannole) (mm) between March to August from 2015 to 2020 for (**a**) Nardò meteorological station—Ionian Coast and (**b**) Otranto meteorological station-Adriatic coast.

**Figure 2:**
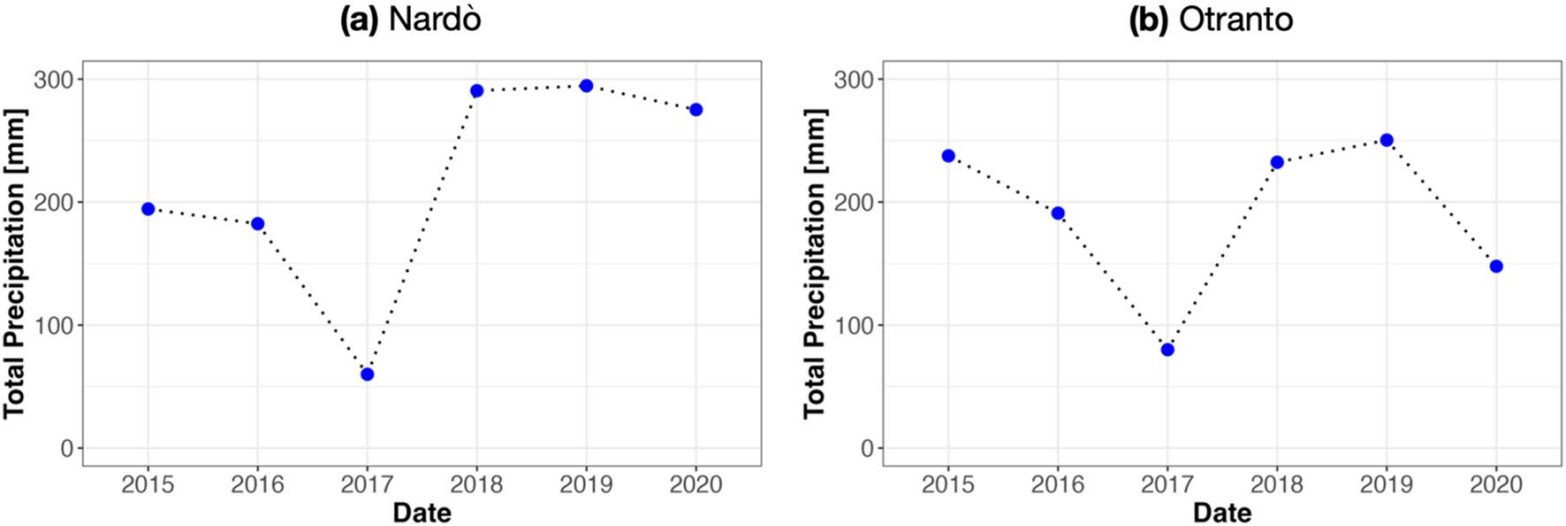
Total spring–summer precipitation (mm) from 2015 to 2020 for (**a**) Nardò meteorological station—Ionian Coast and (**b**) Otranto meteorological station—Adriatic coast.

Meteo-data were collected from the Nardò meteo-station, close to fields of Nardò A, Nardò B, and Galatone, and from the Otranto one, which is close to Cannole. The total precipitation data shown in Figures 1 and 2, refer to the spring-summer season (from March to August) of the years 2015–2020. Expert agronomists report that such month-interval can be useful to understand plant stress conditions. They explain that meteo-events such as heat waves or heavy precipitation in April–May, when young leaves and flowers appear in olive trees, may induce plant stress^31,32^. Specifically, Fig. 1a, b show the total monthly precipitation (March–August) for each year, while Fig. 2a, b report the total precipitation during the spring and summer seasons, on the Ionian and Adriatic coasts, respectively. Figure 1a, b evidence a significant precipitation decrease in March and a slight increase in May on both sides of the coast in the period 2015-2020. Such Figures evidence heavy precipitations in July and August, occurring mainly on the Ionian coast in 2019 and 2020. In this area, due to the compactness of soil following dry periods, these events may have induced flooding.

**Figure 3:**
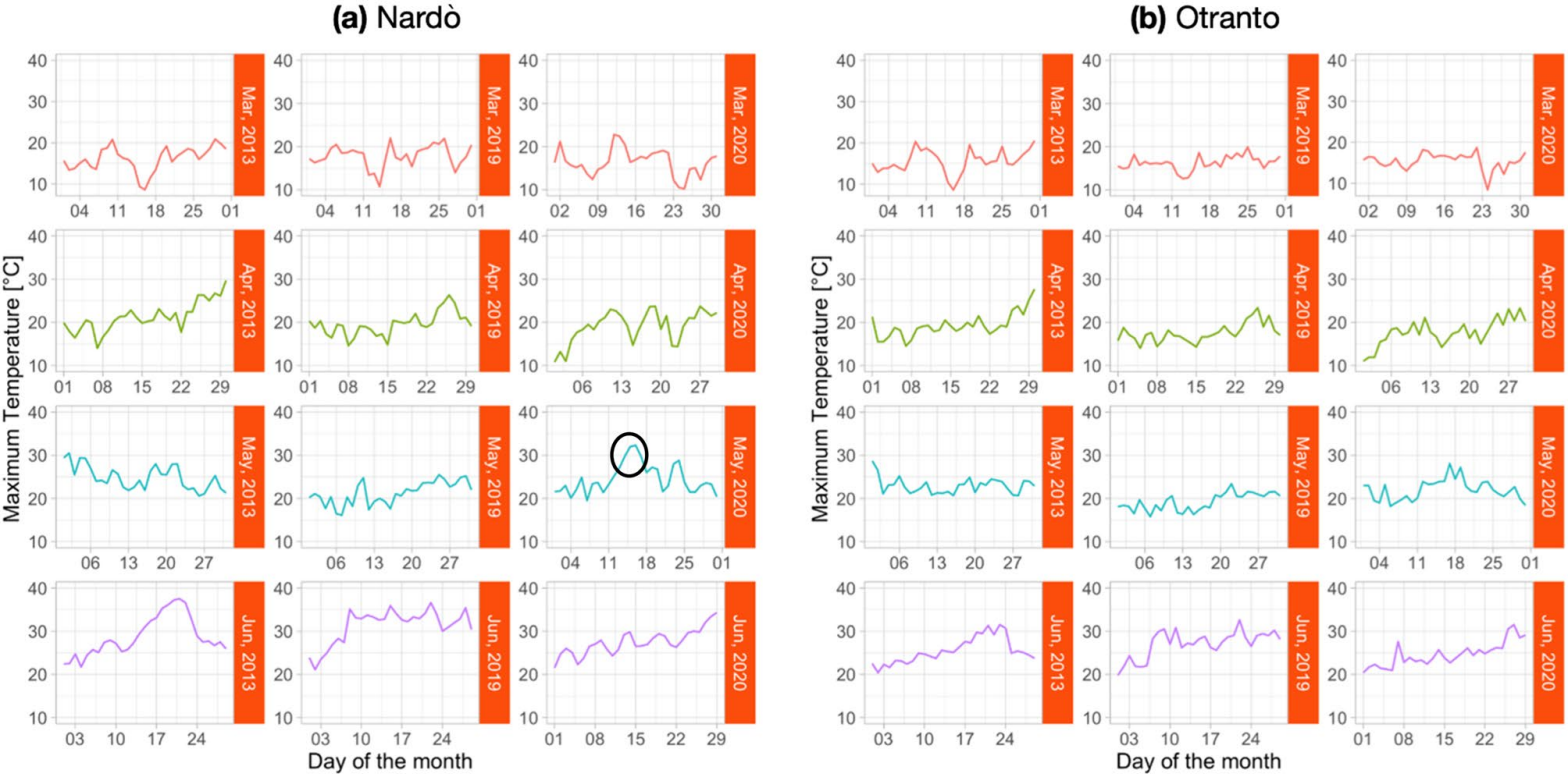
Maximum daily temperature values for 2013, 2019 and 2020, (**a**) Ionian coast; (**b**) Adriatic coast.

Daily maximum temperature values are plotted from March to June, for the years 2013, 2019 and 2020 in Fig. 3. This Figure shows that the Ionian coast experienced higher temperature values (Fig. 3a) than the Adriatic coast (Fig. 3b). This occurrence was more evident in May and June, 2019 and 2020. On the Ionian coast, the mean month temperature was about 23°, but the meteo-station registered temperatures higher than 30° for three consecutive days in May 2020. The black circle in Fig. 3a, third line, shows the heat wave.

### Satellite data: S-2 and Pléiades imagery

For the period 2015–2020, an inter-annual time series of S-2A/2B imagery was freely downloaded from the EarthExplorer portal^33^.

Only 36 images, acquired in July and August, with less than 10% cloud coverage on the full tile (T33TYE from R036 orbit) were investigated (Table 2). S-2 Level-1C products, consisting of geometrically orthorectified and calibrated to Top Of Atmosphere (TOA) reflectance values, were downloaded. As suggested in^34^, the Level-2A surface reflectance product was generated from level-1C by using the Sen2Cor processor for atmospheric correction. The three S-2 atmospheric bands (B1, B9 and B10) at 60 m spatial resolution were excluded from our analysis.

**Table 2:** Acquisition dates for the inter-annual S-2 time series analysed.

Year	Month	S-2 for all fields	Pléiades for each experimental field
2013	July		
	August	None	**18th** Cannole
2015	July	15th–25th	
	August	24th	
2016	July	19th–29th	
	August	28th	
2017	July	4th–14th–24th	
	August	13th–23rd	
2018	July	4th–9th–14th–19th—24th–**29th**	**29th** Galatone, Nardò
	August	**8th**–13th–18th–28th	**6th** Cannole
2019	July	4th–9th–14th–24th–29th	
	August	23rd–28th	
2020	June		**26th** Cannole
	July	**3rd**–8th–28th	**2nd** Galatone, Nardò
	August	12th–17th–27th	

Available closest acquisition dates of both S-2 and Pleiades images are evidenced in bold.

For each experimental field, Pléiades images were selected at the available closest dates to S-2 acquisitions (Table 2). The temporal distance between the acquisitions was a maximum of 7 days. An older Pléiades image from 2013 was also included. For each date, the four pan-sharpened (0.5 m) bands were analysed.

#### S-2 and Pléiades data pre-processing

From S-2 data, the indices listed in Table 3 were analysed to detect changes induced by the restoration action at field-scale. The selected indices result either sensitive to canopy structure/pigment concentration or recommended by^27^ as useful for *Xfp* monitoring from Sentinel-2 data. As well known, the Normalized Difference Vegetation Index (NDVI) is the widest used vegetation index since it can be employed as a proxy of many vegetation variables. The Optimized Soil Adjusted Vegetation Index (OSAVI) can maximize the reduction of soil effects in the vegetation signature. The Normalized Difference Red-Edge (NDRE) includes the Red-Edge portion of the S-2 spectrum, while the Atmospherically Resistant Vegetation Index (ARVI) can reduce atmospheric effects in the vegetation signature.

**Table 3:** Spectral vegetation indices analysed.

Name	Formula
**NDVI** Normalized difference vegetation index	$\frac{{R}_{NIR\left(B8\right)}-{R}_{Red\left(B4\right)}}{{R}_{NIR\left(B8\right)}+{R}_{Red\left(B4\right)}}$
**NDRE** Normalized difference Red-edge	$\frac{{R}_{NIR\left(B8A\right)}-{R}_{RedEdge1\left(B5\right)}}{{R}_{NIR\left(B8A\right)}+{R}_{RedEdge1\left(B5\right)}}$
**OSAVI** Optimized soil adjusted vegetation index	(1 + 0.16)$\frac{{R}_{NIR\left(B8\right)}-{R}_{Red\left(B4\right)}}{{R}_{NIR\left(B8\right)}+{R}_{Red\left(B4\right)}+0.16}$
**ARVI** Atmospherically resistant vegetation index	$\frac{{R}_{NIR\left(B8\right)}-\left[{R}_{Red\left(B4\right)}-\left({R}_{Blue\left(B2\right)}-{R}_{Red\left(B4\right)}\right)\right]}{{R}_{NIR\left(B8\right)}+\left[{R}_{Red\left(B4\right)}-\left({R}_{Blue\left(B2\right)}-{R}_{Red\left(B4\right)}\right)\right]}$

R = Reflectance signal in the spectral range considered: the corresponding S-2 bands are reported in brackets.

The HR S-2 indices were stacked as a data-cube of inter-annual time-series at the native 10 m spatial resolution. For each image, the pre-processing consisted of cropping and spectral index extraction at the native spatial band resolution. Once evaluated, from B5 and B8A at 20 m (Table 3), only the NDRE index was resampled at 10 m through bilinear resampling.

The Pléiades images were pre-processed for converting the Digital Numbers in Top-of-Atmosphere (TOA) reflectance values. Such values can guarantee reduction of inter-scene variability across time and space when clear scenes are available^35^. The SWIR-Cirrus (Band 10) and the “Coastal aerosol” (Band 1) bands available for S-2 can be useful for a robust atmospheric correction, but they are not available for Pléiades. Hence, the atmospheric correction of Pléiades images would have required in-field ancillary data both at several locations within the study area and at the same time of satellite acquisitions. Since in-field data were not available, this correction was not applied in order not to generate an ill or poorly posed issue^35^.

The S-2 and Pléiades data were not utilized as a unique dataset but employed as two separate datasets. Specifically, S-2 and Pléiades were used separately for field-scale and tree-scale analysis, respectively. From Pléiades images, only NDVI and OSAVI indices were extracted among the ones from S-2 (Table 3), due to the lack of the specific band required for the NDRE (Red-Edge band) implementation.

### Multi-scale analysis

The steps of the multi-scale analysis carried out in our study include:

- *Field-scale analysis with HR S-2.* For each study area, the spatial average was computed for each spectral index (Table 3) over all the pixels in the field at each date. Following this, the temporal average was plotted over all the July and August images in the period 2015–2020. Finally, a detailed comparison of index trend values was carried out between these months, for each year. The aim was to evaluate the index variability due to both treatments and rain precipitation in the same months. The study areas include all the treated and untreated fields selected (Table 1).
- *Correlation of HR data with temperature and rain data, from the meteo-stations.* For each index, the analysis focused on the time series of mean values over 2015–2020. The Pearson correlation index was used within the R studio environment^36^.
- *Tree-scale analysis with VHR data.* First, the classification of Pléiades images was carried out for tree crowns extraction through an object-based algorithm. The classification ruleset used as input NDVI values, the area of objects and the first order entropy feature from the green band. The latter was used to discriminate between high and low vegetation^37,38^. To compute entropy, the window size used was optimized for matching the scale of heterogeneity related to variations in object vegetation height, distribution and structure. First, a threshold was applied on the pixel NDVI values to discriminate vegetated from non-vegetated pixels. Next, a threshold on the entropy values discriminated low from high vegetation among the vegetated pixels. Then, an object area rule was adopted to discriminate single-tree crowns within the high vegetation layer. The index values of only pixels from the tree crown objects were finally analysed for both treated and untreated fields over time. For the optimization of bio-fertilizer doses, an additional comparison was carried out between the spectral index responses of each cultivar.
- *Field-scale correlation between atmospherically corrected HR S-2 index values and atmospherically uncorrected VHR Pléiades results*. In our study, S-2 and Pléiades data were utilized as two separate datasets. For these, the same pre-processing procedure was thus not required. Based on this premise, the main objective of the proposed correlation was to verify whether the index trends obtained through the field scale analysis from S-2 were coherent with those obtained from all the tree crowns in Pléiades. We produced the scatter plots between NDVI and OSAVI mean values from S-2 and Pléiades, first, from tree crown pixels only, next, from all the pixels in the field.

## Results

Our study has provided useful indications about the effectiveness of restoration actions to recover infected olive groves. The results of the different methodological steps used for the multi-scale analysis are presented hereafter.

### Field-scale analysis

**Figure 4:**
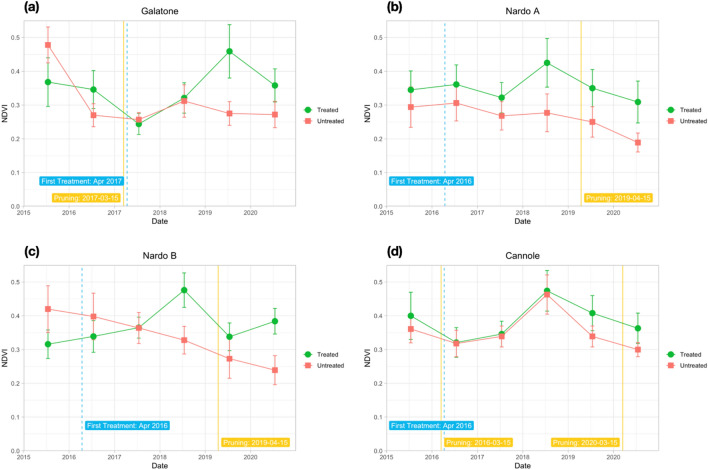
S-2 NDVI trend from each experimental field with or without restoration treatment. (**a**) Galatone; (**b**) Nardò A; (**c**) Nardò B; (**d**) Cannole. Starting of the restoration treatment and pruning dates are indicated. Fields (**a**), (**b**), (**c**) located on the Ionian coast and field (**d**) situated on the Adriatic coast.

The aim of field scale analysis was to evaluate the index variability due to both treatments and rain precipitation in the same months. For Galatone, Nardò A, Nardò B, and Cannole experimental fields, the analysis focused on the temporal trend of NDVI, NDRE, OSAVI, and ARVI field mean values from the July–August S-2 time series in 2015-2020. Similar index trends were obtained for both the treated and untreated fields. NDVI showed always higher values than the other indices. Thus, only the temporal trend of NDVI is reported in Fig. 4, while the trends of other indices are shown in the Supplementary Figures S1, S2 and S3.

As an additional way to compare multitemporal trends, at field-scale, spectral indices of treated and untreated fields were normalized. The index value at the year before the beginning of treatments was used as reference value for index normalization before comparing the relative evolution of both index groups through time. Once normalized, the values observed for treated fields were found always higher than untreated ones.

For the whole period analysed (2015–2020), Fig. 4 shows that before starting bio-fertilizer treatments, NDVI values resulted lower than the ones from untreated fields in both Galatone and Nardò B experimental fields, due to initial worse conditions of the treated fields. After treatments, started in 2016 and 2017 at Nardò B and Galatone, respectively (Table 1), NDVI from the treated fields increased and remained higher than the untreated ones. This trend was confirmed in 2020 even though a general average index value decrease occurred. This decrease might have been due both to tree pruning (Table 1) and a three-day heat wave with temperatures higher than 30° in May. Strong winds were also recorded in the same period. Thus, young leaves, which are more vulnerable to meteo-events, must have suffered from such conditions^33^. The same index decrease was registered in Cannole field, even without the occurrence of heat waves and/or strong winds. For this reason, a quantitative correlation analysis was also carried out between meteo-data (rain and temperature values) and spectral indices (see below, Fig. 10).

**Figure 5:**
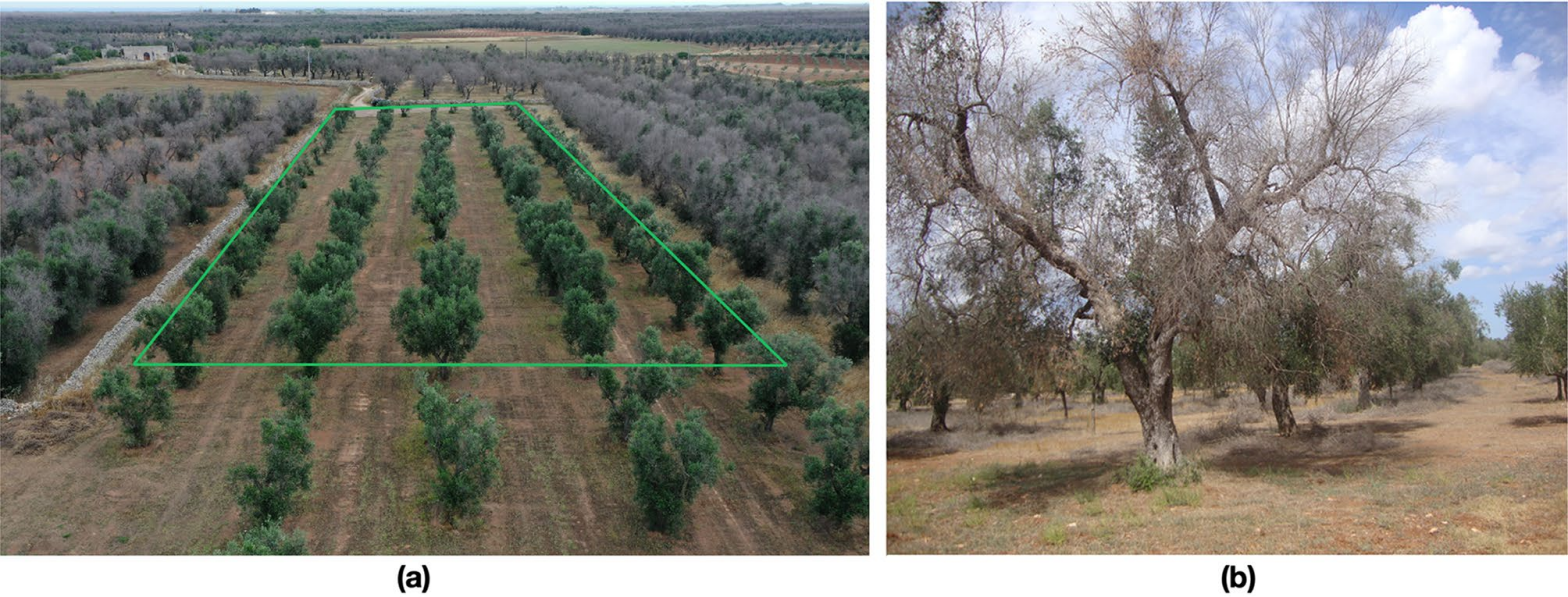
Cannole: (**a**) Image detected from drone in June 2022, after treatments. In green polygon, treated field surrounded by untreated fields; (**b**) tree with *Xfp* symptoms. Oliarola Salentina and Cellina di Nardò cultivars are planted both in treated and untreated fields.

In Fig. 5, a recent drone image (2022) highlights differences between the tree crowns in the Cannole treated field and the surrounding untreated areas. In this field, trees recovered green foliage and became productive owing to treatments.

**Figure 6:**
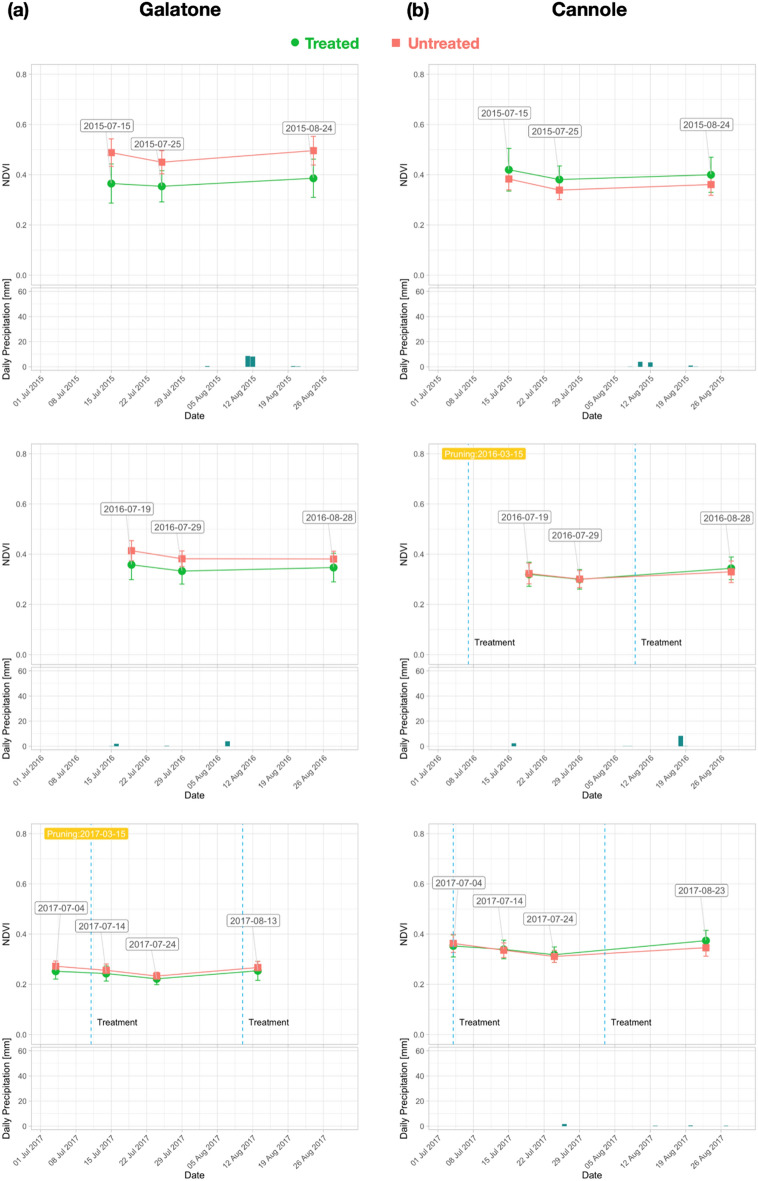
NDVI trend for each year (2015–2017) from S-2 date: (**a**) Galatone; (**b**) Cannole.

**Figure 7:**
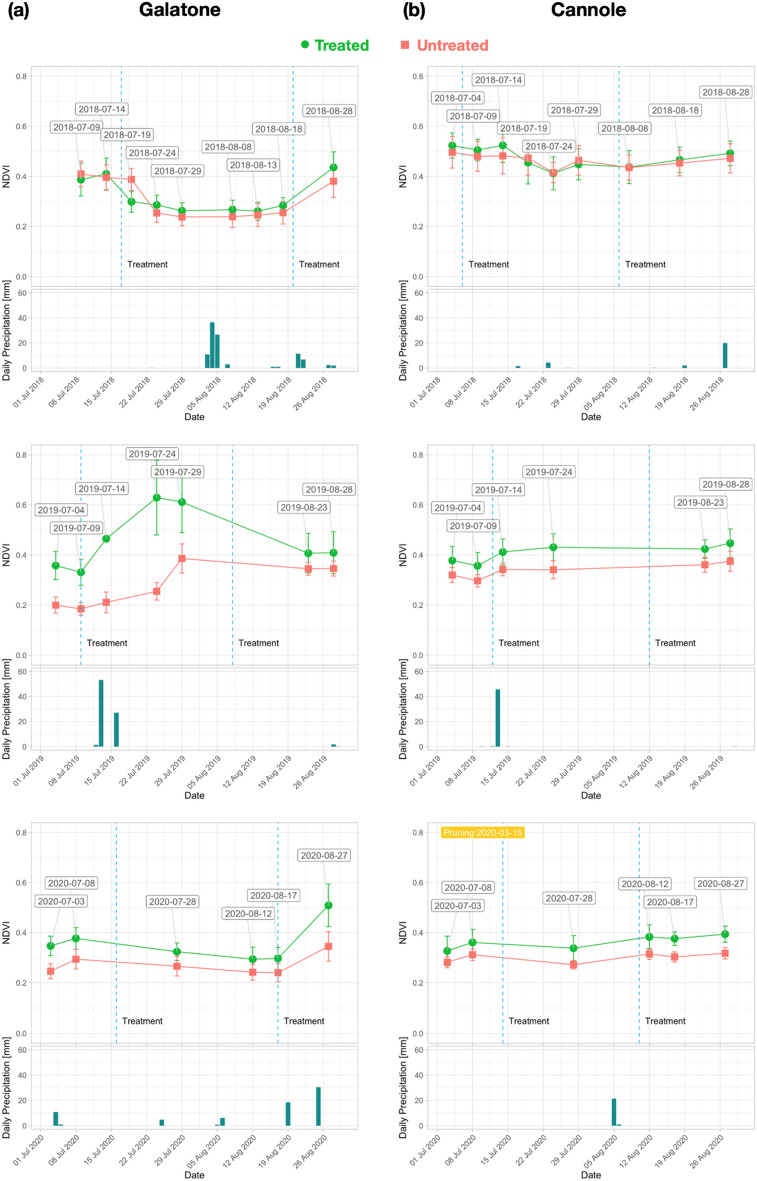
NDVI trend for each year (2018–2020) from S-2 date: (**a**) Galatone; (**b**) Cannole.

**Figure 8:**
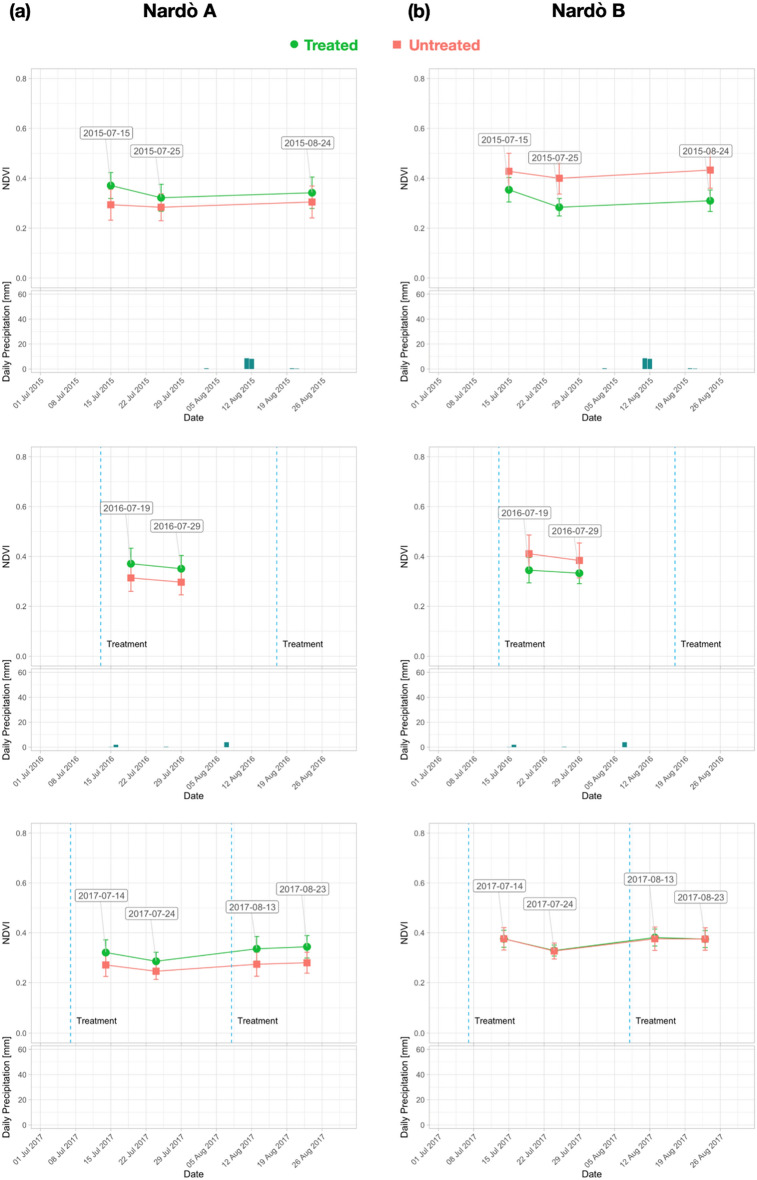
NDVI trend for each year (2015–2017) from S-2 date: (**a**) Nardò A; (**b**) Nardò B.

**Figure 9:**
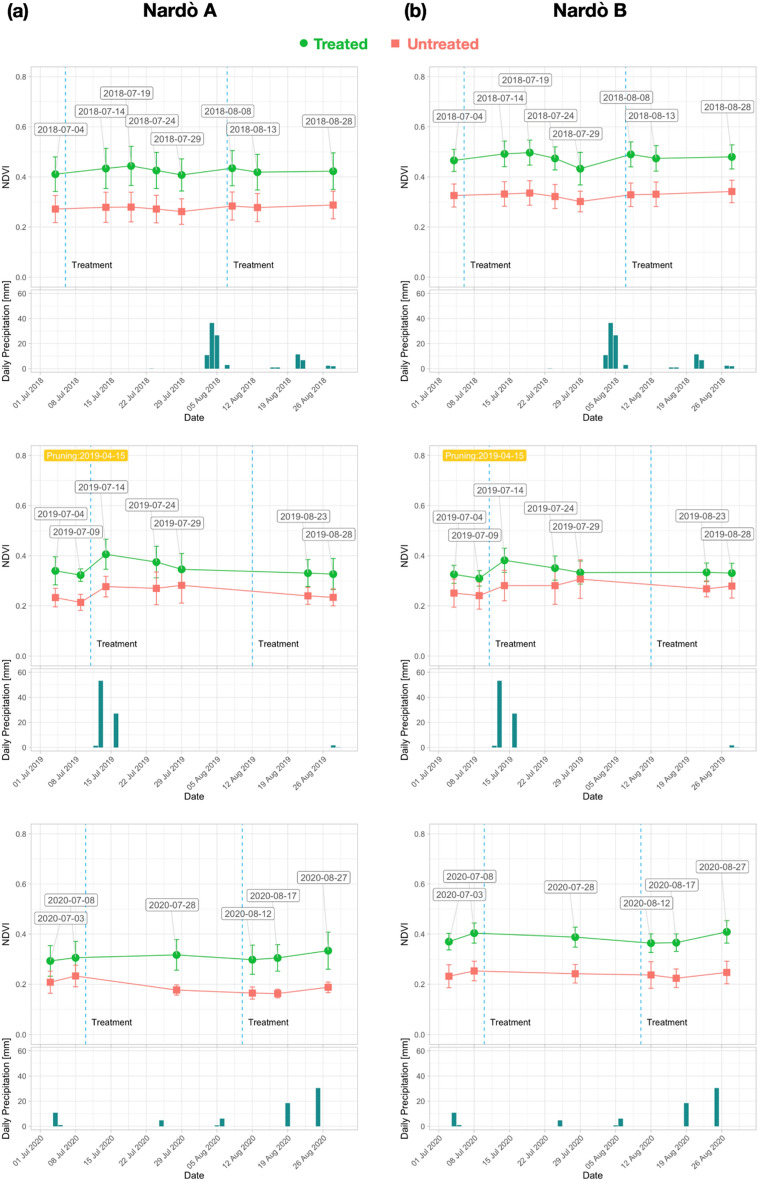
NDVI trend for each year (2018–2020) from S-2 date: (**a**) Nardò A; (**b**) Nardò B.

The yearly NDVI trend analysis is presented in Fig. 6a, b for Galatone and Cannole, respectively, for the years 2015–2017 and in Fig. 7a, b for the period 2018-2020. Data from Nardò A and Nardò B are shown in Fig. 8a, b for the first period and Fig. 9a, b for the second period, respectively. These figures also report daily rain data.

For Galatone (Fig. 6a), as well as for Nardò B (Fig. 8b), the NDVI analysis in the first period indicated higher values for the untreated fields with respect to the fields where treatment had started in 2016 and 2017. Strong water stress conditions can be evidenced by the results from all fields in 2017 (Fig. 6a, b; Fig. 8b). The lowest rain precipitation was recorded in June and August 2017, when NDVI values resulted quite similar for both treated and untreated fields. The treatment impact on the NDVI values began to be evident in the second period, 2018–2020, (Fig. 7a, b; Fig. 8a, b).

### Correlation with meteo-data

**Figure 10:**
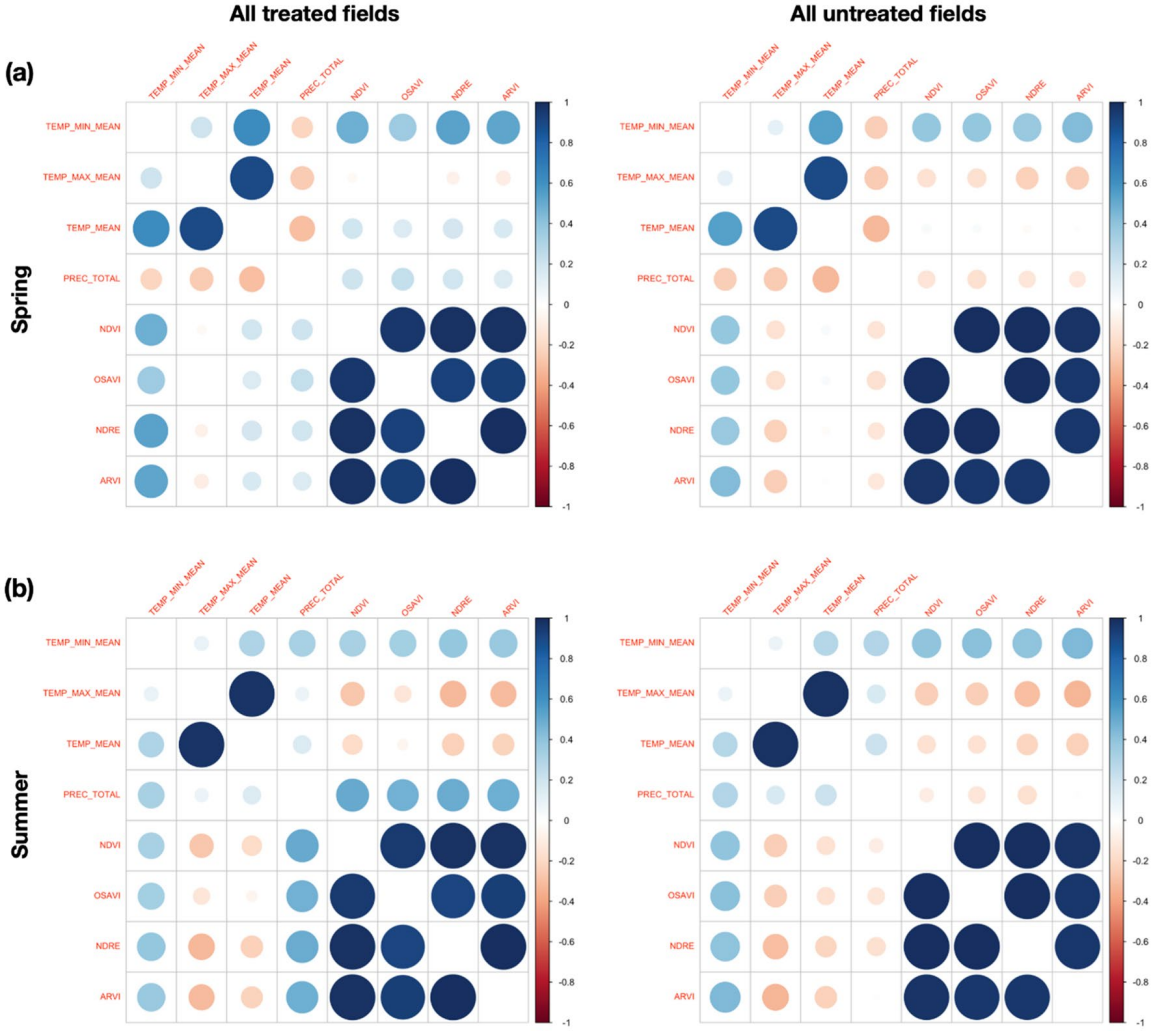
Pearson correlation values between S-2 spectral indices (2015–2020), temperature (min, max, mean values) and total rain precipitation (**a**) from March to May; (**b**) from June to August in all treated and untreated fields.

To evaluate the relationships between all spectral indices and both temperature and precipitation data, a correlation analysis has been carried out using the Pearson index^36^. The values obtained are reported in Fig. 10 for all S-2 spectral indices analysed, collected from March to May and from June to August (2015–2020). These months were the most critical for local vegetation due to water stress. In Fig. 10, the size and colour intensity of each circle represents the correlation strength. Larger and darker circles indicate a higher correlation than smaller and lighter circles. The blue circles represent a positive correlation, while the red ones imply negative values. The correlation between all spectral indices and rain data (PREC_TOTAL) resulted better for treated fields than untreated ones throughout the period 2015–2020. Thus, untreated trees appear less responsive to rain, since their correlation with precipitation appear to be low especially in summer (Fig. 10b).

### Tree-scale analysis with VHR

**Figure 11:**
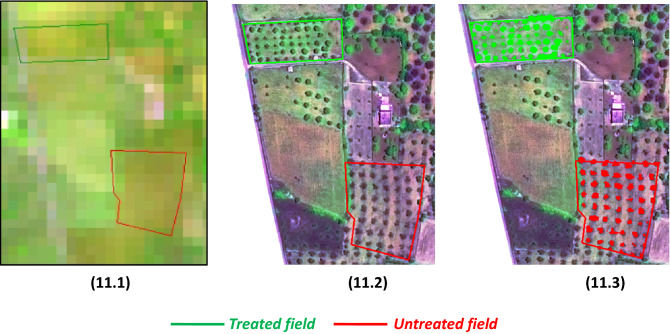
Galatone window images from HR S-2 (7.1), VHR Pléiades (7.2) and VHR automatically classified (7.3). Boundaries of treated (green) and untreated (red) fields are overlaid in the scenes.

To evaluate the different cultivar responses to the bio_fertilizer, the VHR spectral index responses of each cultivar were analysed. To this purpose, the object based automatic classification algorithm, described in the Method section, was applied to the whole Plèiades scenes for tree crown extraction. Figure 11 shows input window images from both S-2 (Fig. 11.1) and Pléiades (Fig. 11.2) images, for Galatone area. Only the object tree crowns of the treated (green) and untreated (red) fields are evidenced in Fig. 11.3 as classification output. To validate the tree crown classification result, a visual photo-interpretation by an independent observer expert was used. For each field and for each image, two tree crowns were randomly selected and used as reference samples. The output Overall Accuracy (OA) was 99.8%.

**Figure 12:**
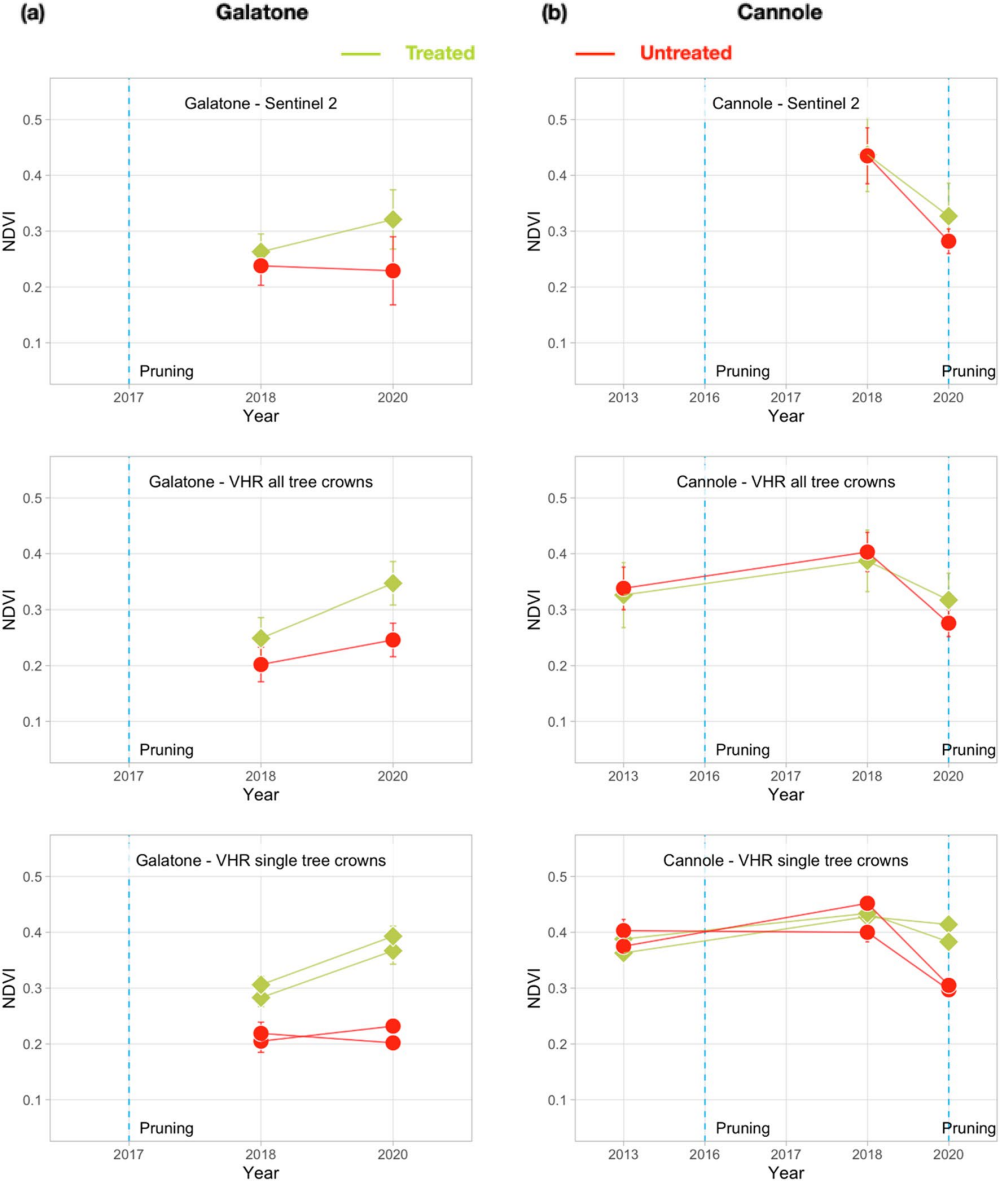
NDVI mean value for Galatone (Ionian coast, column **a**) and Cannole (Adriatic coast, column **b**) from: all S-2 pixels in the field (the first line); only all Pléiades tree crown pixels in the fields (second line); Pléiades tree crown pixels of different sample trees (third line).

The values obtained for both OSAVI and NDVI were similar, thus, only NDVI values are reported hereafter. Figure 12a, b show the NDVI trends obtained separately from S-2 and Pléiades, for Galatone and Cannole, respectively. For treated and untreated fields, Figure 12 includes three lines: the first shows S-2 NDVI values; the second reports the Pléiades mean NDVI values obtained from only all the tree crown pixels in the fields; the third presents the Pléiades NDVI values obtained for only two sampled tree crowns in the fields. Figure 12a, b evidence that, NDVI values from both Pléiades and S-2 images have similar trends, with higher values in the treated fields than in the untreated ones.

Specifically, for Galatone, the NDVI values of only all the tree crown pixels evidence an increase from 2018 to 2020 in the treated field (second line, Fig. 12a). Instead, in the untreated field, a quite constant trend can be observed. This difference could be appreciated in the single-tree crown analysis (third line, Fig. 12a), which shows better the treatment impact on the trees. In Cannole, where treatments had started in 2016, NDVI values from treated and untreated fields remained similar up to 2018. This could have been due to later spreading of *Xfp* on the Adriatic side with respect to the Ionian coast. In 2020, a decrease of all the values could be observed (second and third lines, Fig. 12b). This may have been due to both tree pruning (March), and low rainfall (about 5 mm) recorded in June, before the Pléiades acquisition (June 26th). Even though the tree pruning had occurred, the NDVI values from the treated field remained higher than the untreated one.

**Figure 13:**
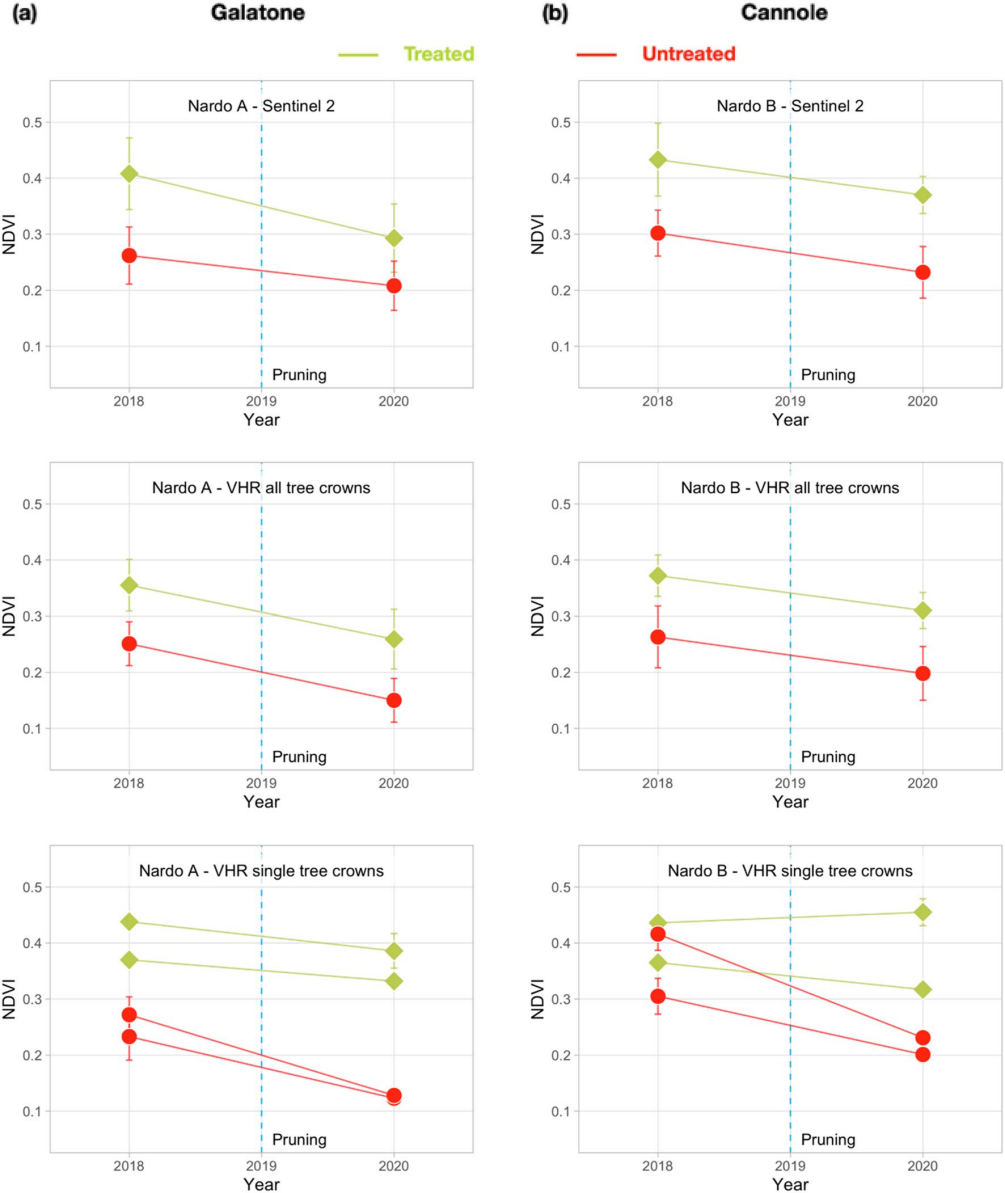
NDVI mean value for test fields, Nardò A (column **a**) and Nardò B (column **b**), same Ionian coast, from: all S-2 pixels in the field, first line; all tree crown pixels only in VHR Pléiades images, second line; tree crown pixels of different sample trees in VHR images, third line.

The additional analysis carried out for Nardò A and Nardò B treated fields, close to the Nardò meteo-station, is reported in Fig. 13a, b. The findings from these two fields confirm that after treatments, the NDVI values from treated trees, remained higher than those from untreated ones. Even though Pléiades images were not atmospherically corrected, the NDVI trends from Pléiades were compatible with those obtained from S-2, for all the study areas.

**Figure 14:**
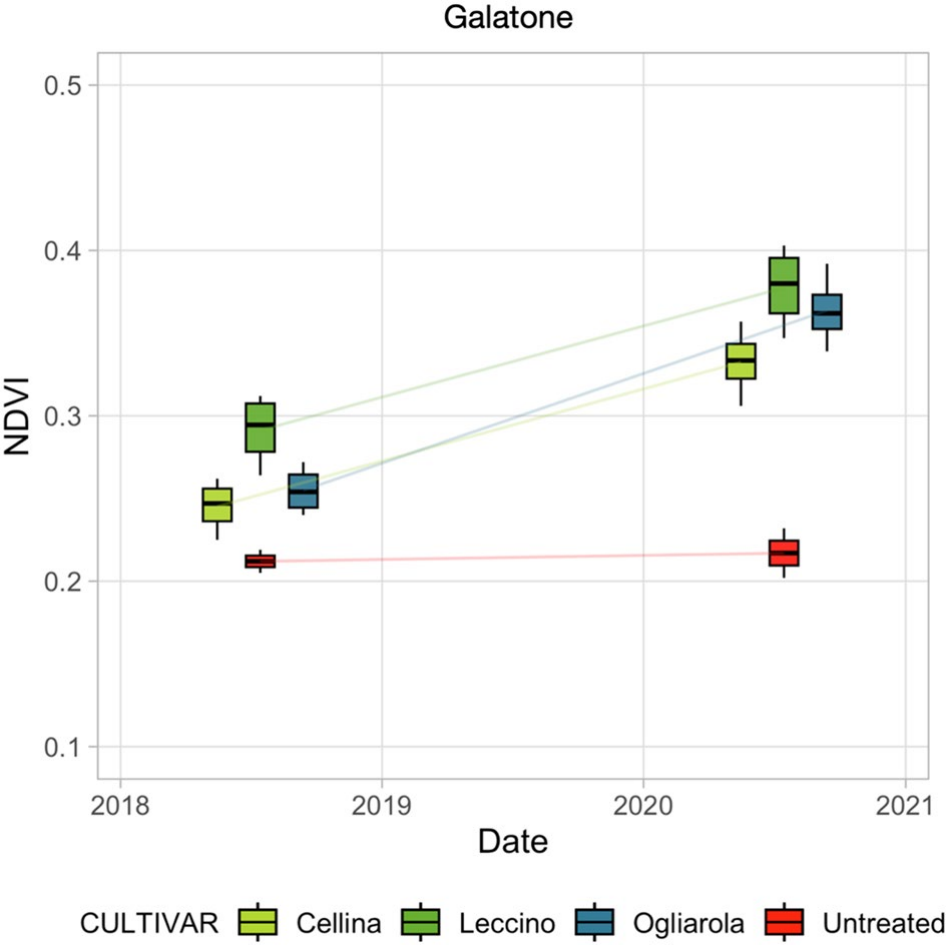
NDVI mean values from different cultivars, i.e., Leccino, Ogliarola Salentina, Cellina di Nardò, with respect to untreated trees in Galatone.

The analysis of VHR data was very useful for evidencing the response of trees from different cultivars to the bio-fertilizer. As already mentioned (Methods), the treated Galatone field includes three different cultivars, i.e. Leccino, Ogliarola Salentina and Cellina di Nardò. Figure 14 reports the NDVI values of four different trees per each cultivar found in the treated Galatone field. For comparison purposes, the figure includes also two sample trees from each of the untreated fields close to Galatone, but for each of these samples the specific cultivar information is not available.

In 2018, before treatments, NDVI values from Leccino resulted slightly higher than the ones from other cultivars in Galatone (Fig. 14). As reported in^13^, Leccino proved more resilient to *Xfp.* However, after treatments, Ogliarola Salentina seems to respond better than both Leccino and Cellina cultivars. In 2020, Ogliarola Salentina reached NDVI values similar to the Leccino ones. The data from untreated trees remained lower than the ones from all treated cultivars. Cellina appeared to be the least responsive to treatments in terms of NDVI.

### Correlation of HR and VHR index trends at field-scale

To compare spectral index trends between HR and VHR imagery, a correlation analysis was carried out at field scale.

**Figure 15:**
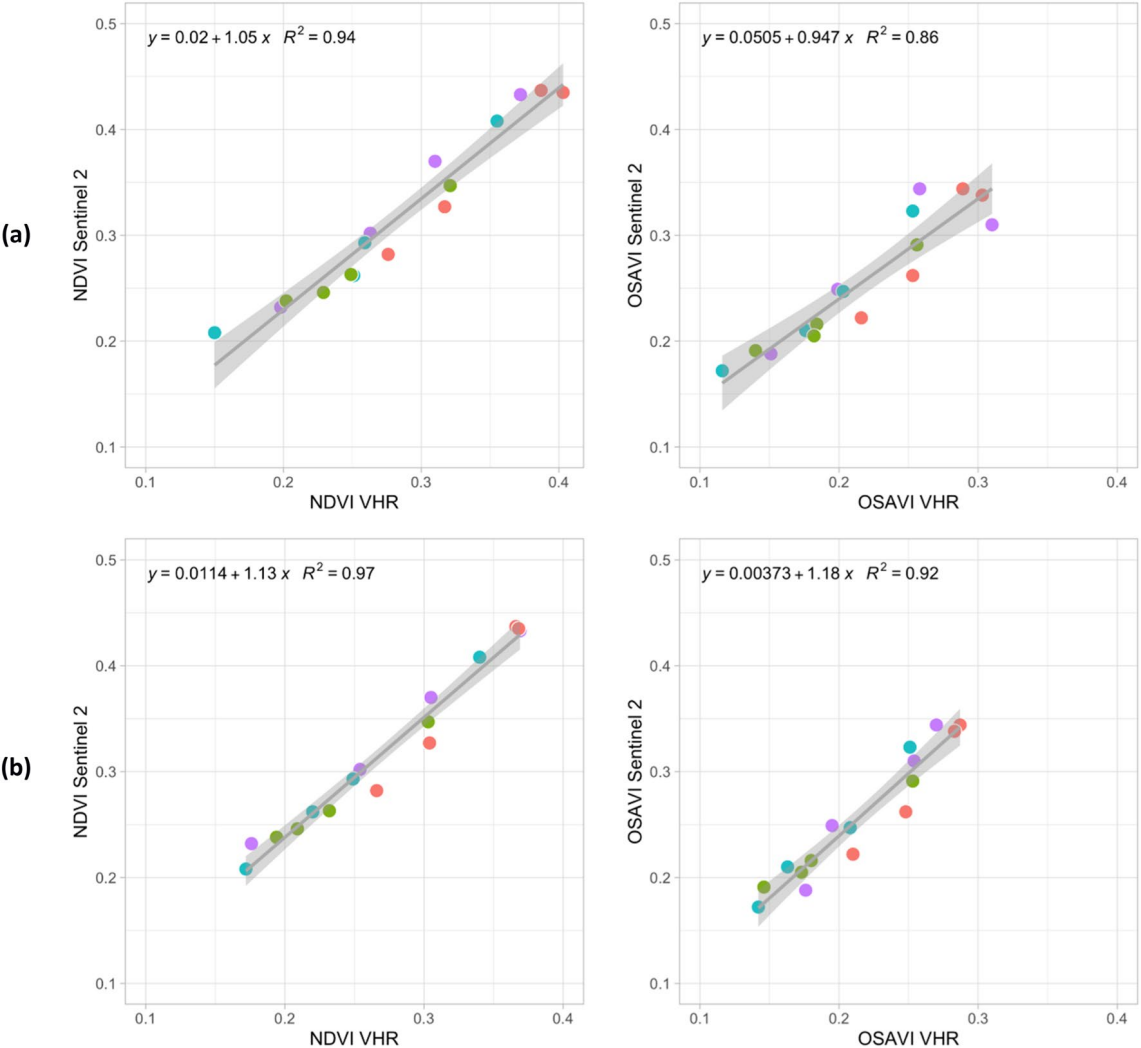
Scatterplot of NDVI and OSAVI field mean values from S-2 and (**a**) index mean values from only tree crown pixels from Pléiades images; (**b**) index mean values all over Pléiades pixels from available treated and not treated fields.

For all treated and untreated fields available, all the scatterplots of NDVI and OSAVI values, obtained from atmospherically corrected S-2 and non-corrected VHR Pléiades imagery, are shown in Fig. 15a, b. In particular, the NDVI and OSAVI values from S-2 data are plotted first (15a) with the Pléiades index values from only tree crown objects, then the same indices are presented (15b) with the values of all the Pléiades pixels in the fields. All the scatterplots obtained a good correlation between S-2 and Pléiades indices, with R2 ranging from 0.94 to 0.97 for NDVI and from 0.84 to 0.92 for OSAVI. Moreover, a systematic overestimation of the S-2 index values was obtained with respect to the ones from only tree crown pixels in Plèiades imagery. Even though VHR data were not atmospherically calibrated, high correlation values were found, at field scale, between S-2 and Pléiades indices (Fig. 15b). This finding confirms that the field analysis with S-2 is coherent with the tree crowns analysis from Pleiades data.

## Discussion

The present paper discusses an application of multi-resolution satellite data to evaluate the effectiveness of a restoration action in olive groves affected by *Xfp* in Salento, Apulia Southern Italy. Restoration actions of olive agro-ecosystems are of critical importance for the entire Mediterranean basin, not only for supporting local economies but also to increase the area resilience to climate changes. The number of studies demonstrating that olive agro-ecosystem can reduce net emissions of greenhouse gases and land degradation processes has been expanding in recent years^15,19,39–41^. These studies appear to justify the efforts for recovery actions of olive groves affected by *Xfp* in the Mediterranean area.

Recent literature on EO monitoring schemes of restoration actions has mainly focused on forest ecosystems^42–44^, whereas, at the best of our knowledge, no papers have been presented on EO monitoring of *Xfp* restoration actions. Our study has focused on the effectiveness of the Dentamet bio-fertilizer. This compound was chosen after a preliminary evaluation of its bactericidal activity towards *Xylella fastidiosa* and for its significant systematicity within the xylem tissue of olive trees^22^. The same bio-fertilizer was verified also effective towards the pathogenic fungus *Plenodomus tracheiphilus*, causal agent of citrus “mal secco”^45^. Dentamet was also found effective for controlling some insects, namely *Halyomorpha halys* (i.e., brown marmorated stink bug), and *Bactrocera oleae* (i.e., olive fruit fly), through the suppression of the bacterial symbionts that occur on the egg surface (i.e., symbiotic control of pests)^46,47^.

To evaluate both tree physiology and status after treatments, spectral indices were used as proxy of in-field verification. Spectral indices were analysed in treated and untreated fields of Salento at two different spatial scales from S-2 (10 m) and Pleiadès (2 m) data. The analysis was performed in July and August (2015–2020 period) when the symptoms induced by *Xfp* were more evident and dry grass on the soil background did not contribute to the crown index values. The results of our study indicated that the spectral index values from HR and VHR satellite images, at field scale and tree scale, respectively, resulted higher than the ones of the untreated trees. Specifically, from the multi-temporal HR S-2 time series of June and August, the analysis of NDVI, NDRE, OSAVI, and ARVI evidenced that all indices had a similar trend. However, NDVI reported higher values (Figure 4, S1, S2, S3).

In our work, the starting ARVI values in 2015 (Figs. 6 and 8) revealed that Galatone, Nardò A and Nardò B fields, facing the Ionian coast, were initially in worst condition with respect to the treated field of Cannole, located on the Adriatic coast. This might be due to the fact that *Xfp* symptoms had appeared years earlier on the Ionian Coast than on the Adriatic coast (2008–2009 and 2016, respectively)^20^.

The authors of a recent paper^27^ focusing on the *Xfp* spreading evidenced that ARVI and OSAVI indices can provide higher performance than NDVI for evaluating both the *Xfp* disease and its incidence severity. However, their focus was on *Xfp* spreading, while our main interest was on the effectiveness of the bio-fertilizer treatments for the restoration of trees affected by this pathogen. To this purpose, the trend of only NDVI seems adequate for our investigation.

To evaluate how long it had taken for trees to respond to treatments, as well as to evidence the possible impact of meteo-events on the spectral index trends, the yearly time-series was also analysed. The findings (Figs. 6 and 7) revealed that in all treated fields investigated, all spectral index values started to increase after two years from the beginning of the treatments with the bio-fertilizer. For the Cannole and Galatone areas, the values for treated fields with respect to their neighbour untreated ones were found to be similar in the years when the treatments had started (2016 and 2017, respectively). In addition, the drought event occurred in 2017 may have slowed down plant recovery (Fig. 6). Following almost two years of tree treatments, the NDVI, OSAVI, NDRE and ARVI index values of the trees resulted higher than the untreated ones. After three years of treatments, the yield of all olive trees (Ogliarola Salentina, Leccino and Cellina di Nardò) was the same as the one obtained before the *Xfp* epidemic, i.e., 25-30 q.ls/ha^23^.

The index values seem to be affected also by meteo-data (Figs. 6, 7, 9a, b), in agreement with previous studies^48,49^. These studies have shown that NDVI follows rainfall with varying time lags dependent on environmental factors, such as soil and vegetation types. On the one hand, the bio-fertilizer treatment might have developed plant resilience to the heat wave which occurred on the Adriatic coast in May 2020. This is in agreement with the evidence reported in^32^, where the transient increases in temperature above normal average values (heat wave) could have caused a marked impact upon the photosynthetic and stomatal physiology of plants. On the other hand, an increase of rain events induced an increase of the vegetation index values in the treated olive trees (Fig. 7). By contrast, the untreated trees that showed extensive sign of twig die-back seemed less responsive to precipitations. Although olive trees are a well-adapted species against drought, considerable energy resources are needed to protect them from water stress. When the soil is saturated with water, for some days, anaerobic conditions can prevail, inducing root hypoxia and additional stress in olive trees^20^. This process can induce reduction of plant growth and productivity, which in turn may be reflected in low vegetation index values^50^. When the spectral indices were correlated with temperature and rain precipitation (Fig. 10), high Pearson index values indicated a relationship between rainy events, occurring from March to May and from June to August, and the spectral indices analysed (2015–2020). Thus, the trend of Pearson correlation values could be potentially utilized as an indicator for either the abnormal status of *Xfp* severely attacked trees or plant recovery on large scale.

To monitor the effectiveness of treatments, over large areas, the correlation between freely available S-2 data and more costly VHR data was carried out. For this analysis, only NDVI and OSAVI indices were used from VHR Pléiades images. Even though not atmospherically corrected, the trend of Pléiades vegetation indices showed a response to the treatments similar to the one obtained from field scale analysis from calibrated S-2 data. The high correlation values obtained (Fig. 15) confirms that the field analysis with S-2 is coherent with the tree crowns analysis from Pleiades data. Thus, the former images may be used instead of more costly VHR Pléiades data.

To evaluate the treatment responsivity of different cultivars, VHR tree scale analysis was carried out. The findings obtained revealed that all cultivars seemed to be able to respond effectively to the treatments. In particular, Ogliarola Salentina and Leccino cultivars responded better to the treatments, with higher NDVI and OSAVI values than Cellina di Nardò (Fig. 14). The combination of water deficit and Xfp infection may have caused a relevant reduction of genes related to drought response in Cellina di Nardò during summer as evidenced in^51^. In a more recent study, based on phenomics approach for the screening of susceptible/resistant genotypes to *X. fastidiosa*, severe symptoms of desiccation were observed for *Xfp*-infected plants of Cellina di Nardò, 18 months after vector transmission, while plants of Leccino remained symptomless^14^.

The findings of this study are in agreement with the results obtained through in-field quantitative real-time PCR^12^ and nuclear magnetic resonance (H-NMR) based metabolomic analyses^25^. In the experimental fields analysed, both techniques revealed a significant reduction of *Xfp* cell density within the leaves and a re-programming of some metabolic pathways toward a normal tree physiology upon the treatments with Dentamet. It seems worth noting that the olive tree age could be important in the response to the treatment since young trees are more reactive to the compound. Up to now, the experimentation has only been conducted on young trees (under 100 years). The results obtained have encouraged the design of Dentamet experimentation on fields with centenary/millennial olive trees. These trees characterize most of the landscape of Southern Apulia. To be effective, treatments should be applied to trees having at least 50% of the crown not wilted. This in order to allow the compound to penetrate within the xylem of the leaves and circulate.

In the study areas analysed, all the affected trees, including the susceptible varieties, recovered completely after the treatments. Thus, the proposed multitemporal and multi-scale analysis with EO data could support both the monitoring, the evaluation and/or the selection of adequate olive agro-system recovery actions. As evidenced by^32^, such selection could be useful to foster transition towards sustainable agriculture in the Mediterranean areas and reduce the unsustainable use of pesticides, according to the European Green Deal Strategy^52^.

## Conclusions

The present paper seems to respond to the need of quantitative measurements from EO data for validating the effectiveness of restoration actions for the recovery of the olive-grove ecosystem under *Xylella fastidiosa* subsp. *pauca* attack. All spectral indices analysed in the July and August time series of S-2 images showed higher values in treated fields than in the untreated ones. At field scale, the HR analysis combined with meteo-events, in the whole periods and daily per each year, evidenced that treated olive trees could reduce the bacteria load after two years and become again productive after three years of treatments. VHR data evidenced the response of different cultivars to the Dentamet bio-fertilizer. Due to undergoing climate change and the extension of *Xfp* infection, all the results obtained suggest urgent need for further large scale investigation to reduce the level of *Xfp* infection in the Mediterranean and to accelerate the recovery of olive agro-ecosystems functioning. At the best of our knowledge, no other systematic experimentations with other compounds have been carried out so far.

The use of the Dentamet bio-fertilizer can foster transition towards sustainable agriculture, while EO techniques could support the monitoring of restoration actions and favor new jobs. Restoration actions for *Xfp*, including the one based on Dentamet, are still limited to few experimental areas. EO monitoring of such actions could be of relevance for their promotion not only in Italy, but also in other Mediterranean Countries.

## Supplementary Information

Supplementary Information.

## Data Availability

The authors ensure that all data are presented in the main manuscript and additional supporting files whenever possible. The authors will make materials, data and associated protocols promptly available to others without preconditions.

## References

[1] Saponari M, Boscia D, Nigro F, Martelli GP (2013). Identification of DNA sequences related to *Xylella fastidiosa* in oleander, almond and olive trees exhibiting leaf scorch symptoms in Apulia (Southern Italy). J. Plant Pathol..

[2] Cariddi C, Saponari M, Boscia D, De Stradis A, Loconsole G, Nigro F, Porcelli F, Potere O, Martelli GP (2014). Isolation of *Xylella fastidiosa* strain infecting olive and oleander in Apulia, Italy. J. Plant Pathol..

[3] Picciotti U, Lahbib N, Sefa V, Porcelli F, Garganese F (2021). Aphrophoridae role in *Xylella fastidiosa* subsp. *pauca* ST53 invasion in Southern Italy. Pathogens.

[4] Scortichini M (2022). The epidemiology and control of “olive quick syndrome” in Salento (Apulia, Italy). Agronomy.

[5] Martelli GP (2016). The current status of the quick decline syndrome of olive in Southern Italy. Phytoparasitica.

[6] Kottelenberg D, Hemerik L, Saponari M, van der Werf W (2021). Shape and rate of movement of the invasion front of *Xylella fastidiosa* spp. *pauca* in Puglia. Sci. Rep..

[7] Stokstad E (2015). Italy’s olives under siege. Science.

[8] Scholten, R., Martinez Sanchez, L., Hornero, A., Navas-Cortes, J.A., Zarco-Tejada, P.J., Beck, P.S.A. Monitoring the impact of Xylella on Apulia’s olive orchards using MODIS satellite data supported by weather data. In Proceedings of the 2nd European Conference on *Xylella fastidiosa*, Ajaccio, France, 29–30 October 2019; http://www.efsa.europa.eu/sites/default/files/event/191029-xylella/S6.P1_BECK.pdf.

[9] Delbianco A, Gibin D, Pasinato L, Boscia D, Morelli M, EFSA (European Food Safety Authority) (2023). Update of the Xylella spp. host plant database – systematic literature search up to 30 June 2022. EFSA J..

[10] Marchi G., Rizzo D., Ranaldi F., Ghelardini L., Ricciolini M., Scarpelli I., Drisera L., Goti, E., Capretti P., Surico G. First detection of Xylella fastidiosa subsp. multiplex DNA in Tuscany (Italy). Phytopathologia Mediterranea, 57, 363-364. DOI:0.14601/Phytopatol_Mediterr-24454. (2018).

[11] Schneider, K., van der Werf, W., Cendoya, M., Mouritis, M., Navas-Corte ´s, J.A., Vincent , A., Oude Lansink, A. Impact of *Xylella fastidiosa* subspecies *pauca* in European olives. *PNAS*, **117** (15), 9250–9259. 10.1073/pnas.1912206117 (2022). 10.1073/pnas.1912206117 PMC7196823 32284411

[12] Bollettino Ufficiale della Regione Puglia (BURP), n.139, DELIBERAZIONE DELLA GIUNTA REGIONALE 12 dicembre 2022, n. 1866. Approvazione “Piano d’azione per contrastare la diffusione di *Xylella fastidiosa* in Puglia” biennio 2023–2024, https://burp.regione.puglia.it/documents/20135/2000617/DEL_1866_2022.pdf/45c48e24-2356-789f-2048-85adabcadd72?version=1.0&t=1672141753490 (2022).

[13] Surano, A., Kubaa, R.A., Nigro, F., Altamura, G., Losciale, P., Saponari, M., Saldarelli, P. Susceptible and resistant olive cultivars show differential physiological response to *Xylella fastidiosa* infection. *Front. Plant Sci*., **13**. 10.3389/fpls.2022.968934 (2022). 10.3389/fpls.2022.968934 PMC9530328 36204082

[14] Giampetruzzi, A., Morelli, M., Saponari, M., Loconsole, G., Chiumenti, M., Boscia, D., Savino, V.N., Martelli, G.P., Saldarelli, P. Transcriptome profiling of two olive cultivars in response to infection by the CoDiRO strain of Xylella fastidiosa subsp. pauca. *BMC Genomics*, **17**, 1–18. 10.1186/s12864-016-2833-9 (2016). 10.1186/s12864-016-2833-9 PMC4924284 27350531

[15] Fraga, H., Moriondo, M., Leolini, L., Santos, J.A. Mediterranean olive orchards under climate change: a review of future impacts and adaptation strategies. *Agronomy*, **11**, 56. https://www.mdpi.com/2073-4395/11/1/56 (2021).

[16] Chamizo-Ampudia A, Sanz-Luque E, Llamas A, Galvan A, Fernandez E (2017). Nitrate reductase regulates plant nitric oxide homeostasis. Trends Plant Sci..

[17] Bateni C, Ventura M, Tonon G (2019). Soil carbon stock in olive groves agroforestry systems under different management and soil characteristics. Agroforest Syst..

[18] Moreno, G., Aviron, S., Berg, et al. Agroforestry systems of high nature and cultural value in Europe: provision of commercial goods and other ecosystem services. *Agroforestry Syst.* **92**(4), 877–891 (2018).

[19] Brilli L, Lugato E, Moriondo M, Gioli B, Toscano P, Zaldei A, Leolini L, Cantini C, Caruso G, Gucci R (2019). Carbon sequestration capacity and productivity responses of Mediterranean olive groves under future climates and management options. Mitig. Adapt. Strat. Glob. Change.

[20] Scortichini, M. The multi-millenial olive agro-ecosystem of Salento (Apulia, Italy) threatened by *Xylella fastidiosa* subsp. *pauca*: a working possibility of restoration. *Sustainability*, **12**, 6700. 10.3390/asu.12176900 (2020).

[21] EFSA, European Food Safety Authority. Scientific opinion on the risk to plant health posed by *Xylella fastidios*a in the EU territory, with the identification and evaluation of risk reduction options. *EFSA Journal*, **13**, 3989. 10.2903/j.efsa.2015.5989 (2015).

[22] Scortichini, M., Chen, J., de Caroli, M., Dalessandro, G., Pucci, N., Modesti, V., L’Aurora, A., Petriccione, M., Zampella, L., Mastrobuoni, F., Migoni, D., Del Coco, L., Girelli, C.R., Piacente, F., Cristella, N., Marangi, P., Laddomada, F., Di Cesare, M., Cesari, G., Fanizzi, F.P., Loreti, S. A zinc, copper and citric acid biocomplex shows promise for control of *Xylella fastidiosa* subsp*. pa*uca in olive trees in Apulia region (Southern Italy). *Phytopathologia Mediterranea*, **57**, 48–72. 10.14601/Phytopathol_Mediterr-21985 (2018).

[23] Tatulli, G., Modesti, V., Pucci, N., Scala, V., L’aurora, A., Lucchesi, S., Salustri, M., Scortichini, M., Loreti, S. Further in vitro assessment and mid-term evaluation of control strategy of *Xylella fastidiosa* subsp*. pauca* in olive groves of Salento (Apulia, Italy). *Pathogens*, **10**, 85. 10.3390/pathogens10010085 (2021). 10.3390/pathogens10010085 PMC7835972 33478174

[24] Girelli, C.R., del Coco, L., Angilè, F., Scortichini, M., Fanizzi, F.P. Olive cultivars susceptible or tolerant to *Xylella fastidiosa* subsp*. pauca* exhibit mid-term different metabolomes upon natural infection or a curative treatment. *Plants*, **10**, 772. 10.3390/plants10040772 (2021). 10.3390/plants10040772 PMC8103516 33920775

[25] Girelli, C.R., Hussain, M., Verweire, D., Oehl, M.C., Massana-Codina, J., Avendano, M.S., Migoni, D., Scortichini, M., Fanizzi, F.P. Agro-active endo-therapy treated *Xylella fastidiosa* subsp.* pauca*-infected olive trees assessed by the first 1H-NMR-based metabolomic study. *Sci. Rep.*, **12**. 10.1038/s41598-022-09687-8 (2022). 10.1038/s41598-022-09687-8 PMC8993878 35396514

[26] Zarco-Tejada PJ, Camino C, Beck PSA (2018). Previsual symptoms of *Xylella fastidiosa* infection revealed in spectral plant-trait alterations. Nature Plants.

[27] Hornero, A., Hernández-Clemente, R., Northa, P.R.J., Beck, P.S.A., Boscia, D., Navas-Cortes, J.A., Zarco-Tejada, P.J. Monitoring the incidence of *Xylella fastidiosa* infection in olive orchards using ground-based evaluations, airborne imaging spectroscopy and Sentinel-2 time series through 3-D radiative transfer modelling. *Remote Sens. Environ.*, **236**. 10.1016/j.rse.2019.111480 (2020).

[28] Poblete T, Camino C, Beck PSA, Hornero A, Kattenbornd T, Saponari M, Boscia D, Navas-Cortes JA, Zarco-Tejada PJ (2020). Detection of *Xylella fastidiosa* infection symptoms with airborne multispectral and thermal imagery: assessing bandset reduction performance from hyperspectral analysis. ISPRS J. Photogramm. Remote. Sens..

[29] Olmos-Trujillo E, Gonzales-Trinitad J, Ferreira HJ, Pacheco-Guerrero A, Buatista-Capetillo C, Avila-Sandova C, Galván-Tejada E (2020). Spatio-temporal response of vegetation indices to rainfall and temperature in a semiarid region. Sustainability.

[30] Pavan., S., Vergine M., ETC, Screening of olive biodiversity defines genotypes potentially resistant to *Xylella fastidiosa. Front. Plant Sci.,* **12**, 10.3389/fpls.2021.723879 (2021). 10.3389/fpls.2021.723879 PMC8415753 34484283

[31] Teskey R (2015). Responses of tree species to heat waves and extreme heat events. Plant Cell Environ..

[32] Haworth, M., Marino, G., Brunetti, C., Killi, D., De Carlo, A., Centritto, M. The impact of heat stress and water deficit on the photosynthetic and stomatal physiology of Olive (*Olea europaea* L.): A case study of the 2017 heat wave. *Plants*, **7**, 76. 10.3390/plants7040076 (2018). 10.3390/plants7040076 PMC6313851 30241389

[33] USGS Portal. https://earthexplorer.usgs.gov/. Accessed on 9 May 2018.

[34] ESA Technical Guide. https://sentinel.esa.int/web/sentinel/technical-guides/sentinel-2-msi/level-2aprocessing. Accessed on 26 March 2018.

[35] Baraldi A, Gironda M, Simonetti D (2010). Operational two-stage stratified topographic correction of spaceborne multi-spectral imagery employing an automatic spectral rule-based decision-tree preliminary classifier. IEEE Trans. Geosci. Remote Sens..

[36] Zoungrana BJB, Conrad C, Amekudzi LK, Thiel M, Da ED (2015). Land use/cover response to rainfall variability: a comparing analysis between NDVI and EVI in the Southwest of Burkina Faso. Climate.

[37] Adamo, M., Tomaselli, V., Tarantino, C., Vicario, S., Veronico, G., Lucas, R., Blonda, B. Knowledge-based classification of grassland ecosystem based on multi-temporal WorldView-2 data and FAO-LCCS taxonomy. *Remote Sens.*, **12** (9) (2020).

[38] Petrou Z, Manakos I, Stathaki T, Mucher C, Adamo M (2015). Discrimination of vegetation height categories with passive satellite sensor imagery using texture analysis. IEEE J. Sel. Topics Appl. Earth Observ. Remote Sens.

[39] Proietti S, Sdringola P, Regni L, Evangelisti N, Brunori A, Ilarioni L, Nasini L, Proietti P (2021). Extra Virgin Olive oil as carbon negative product: experimental analysis and validation of results. J. Clean. Prod..

[40] Gkisakis VD, Volakakis N, Kosmas E, Kabourakis EM (2020). Developing a decision support tool for evaluating the environmental performance of olive production in terms of energy use and greenhouse gas emissions. Sustain. Prod. Consump..

[41] Nieto OM, Castro J, Fernandez E, Smith P (2010). Simulation of soil organic carbon stocks in a Mediterranean olive grove under different soil-management systems using the RothC model. Soil Use Manag..

[42] Liu CC, Chen YH, Wu MHM (2019). Assessment of forest restoration with multitemporal remote sensing imagery. Sci. Rep..

[43] Kim AR, Lim BS, Seol J, Lim CH, You YH, Lee WS, Lee CS (2021). Diagnostic assessment and restoration plan for damaged forest around the Seokpo Zinc Smelter, Central Eastern Korea. Forests.

[44] Meroni M, Schucknecht A, Fasbender D, Rembold F, Fava F, Mauclaire M, Goffner D, Lucchio LM, Leonardi U (2017). Remote sensing monitoring of land restoration interventions in semi-arid environments with a before–after control-impact statistical design. Int. J. Appl. Earth Obs. Geoinf..

[45] Olivieri C, Modica G, Bella P, Dimaria G, Cirvilleri G, Continella A, Catara V (2022). Preliminary evaluation if a zinc-copper-citric acid biocomplex for the control of Plenodomus tracheiphilus causal agent of citrus mal secco disease. Acta Hort..

[46] Gonella E, Orrù B, Alma A (2019). Egg masses treatment with micronutrient fertilizers has a suppressive effect on newly-emerged nymphs of the brown marmorated stink bug Halyomorpha halys. Entomologia Generalis.

[47] Checchia I, Perin C, Mori N, Mazzon L (2022). Oviposition deterrent activity of fungicides and low-risk substances for the integrated management of the olive fruit fly Bactrocera oleae (Diptera, Tephritidae). Insects.

[48] Richard, Y., Poccard. A statistical study of NDVI sensitivity to seasonal and interannual rainfall variations in Southern Africa. *Int. J. Remote Sens.*, **19** (15), 2907–2920. 10.1080/014311698214343 (2010).

[49] Olmos-Trujillo, E., González-Trinidad, J., Júnez-Ferreira, H., Pacheco-Guerrero, A., Bautista-Capetillo, C., Avila-Sandoval, C., Galván-Tejada, E. *Sustainability,* **12***,* 1939. https://doi.or/10.3390/su12051939 (2020).

[50] Brito C, Dinis L-T, Moutinho-Pereira J, Correia CM (2019). Drought stress effects and olive tree acclimation under a changing climate. Plants.

[51] De Pascali M, Vergine M, Sabella E, Aprile A, Nutricati E, Nicoli F, Buja I, Negro C, Miceli A, Rampino P, De Bellis L, Luvisi A (2019). Molecular effects of *Xylella fastidiosa* and drought combined stress in olive trees. Plants.

[52] European Green Deal, https://ec.europa.eu/info/strategy/priorities-2019-2024/european-green-deal/delivering-european-green-deal_en.

