# Supplementary material for: Satellite monitoring of bio-fertilizer restoration in olive groves affected by Xylella fastidiosa subsp. pauca
Source: Sci Rep. 2023 Apr 7;13:5695. doi: 10.1038/s41598-023-32170-x (PMC10082035; doi:10.1038/s41598-023-32170-x)
Supplement: Supplementary file 1 — Supplementary Information. [file 41598_2023_32170_MOESM1_ESM.pdf]

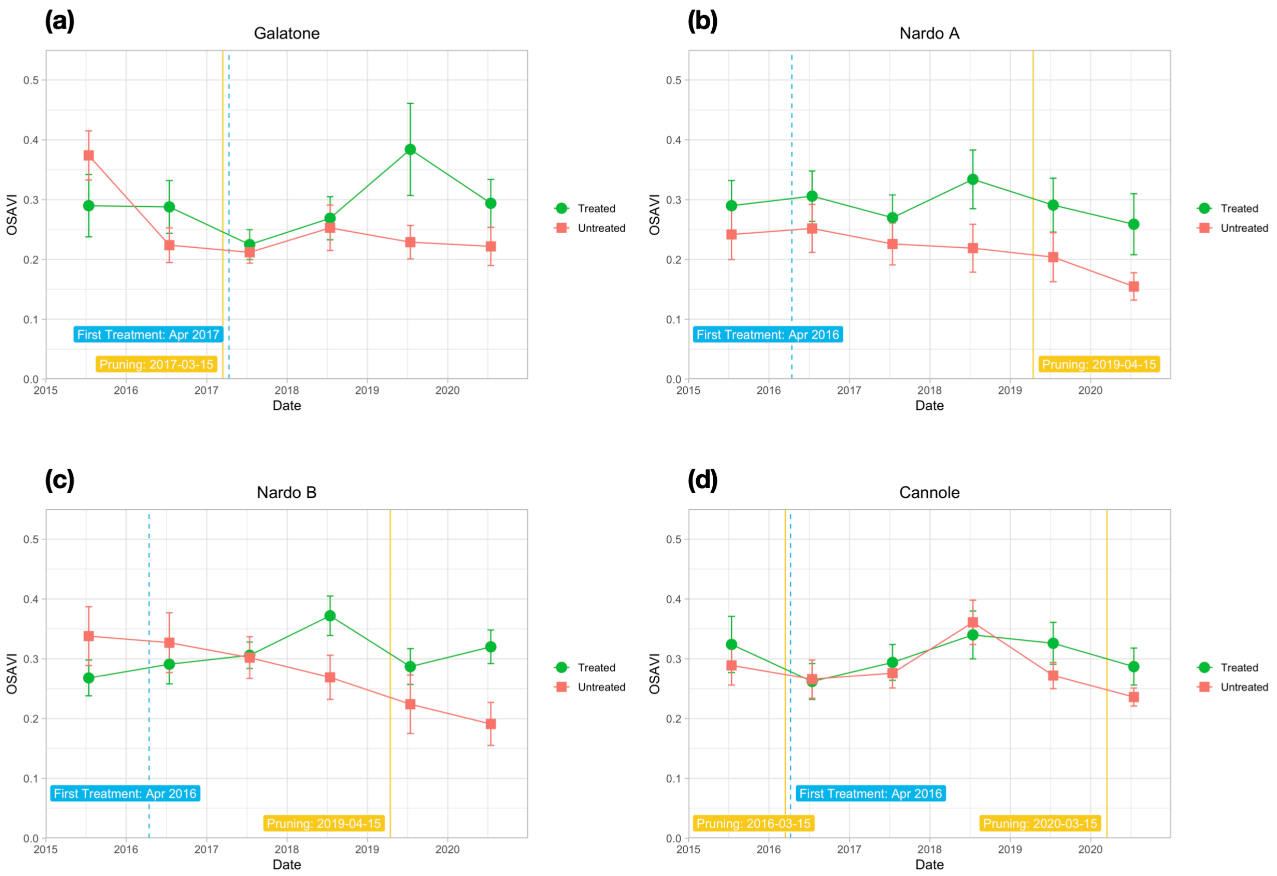

Figure S1. S-2 OSAVI trend from each site in different fields with or without restoration treatment. **(a)** Galatone; **(b)** Nardò_A; **(c)** Nardò_B; **(d)** Cannole. Starting period for the restoration treatment and pruning date are indicated. Sites **(a)**, **(b)**, **(c)** on the Ionian coast and site **(d)** on the Adriatic coast.

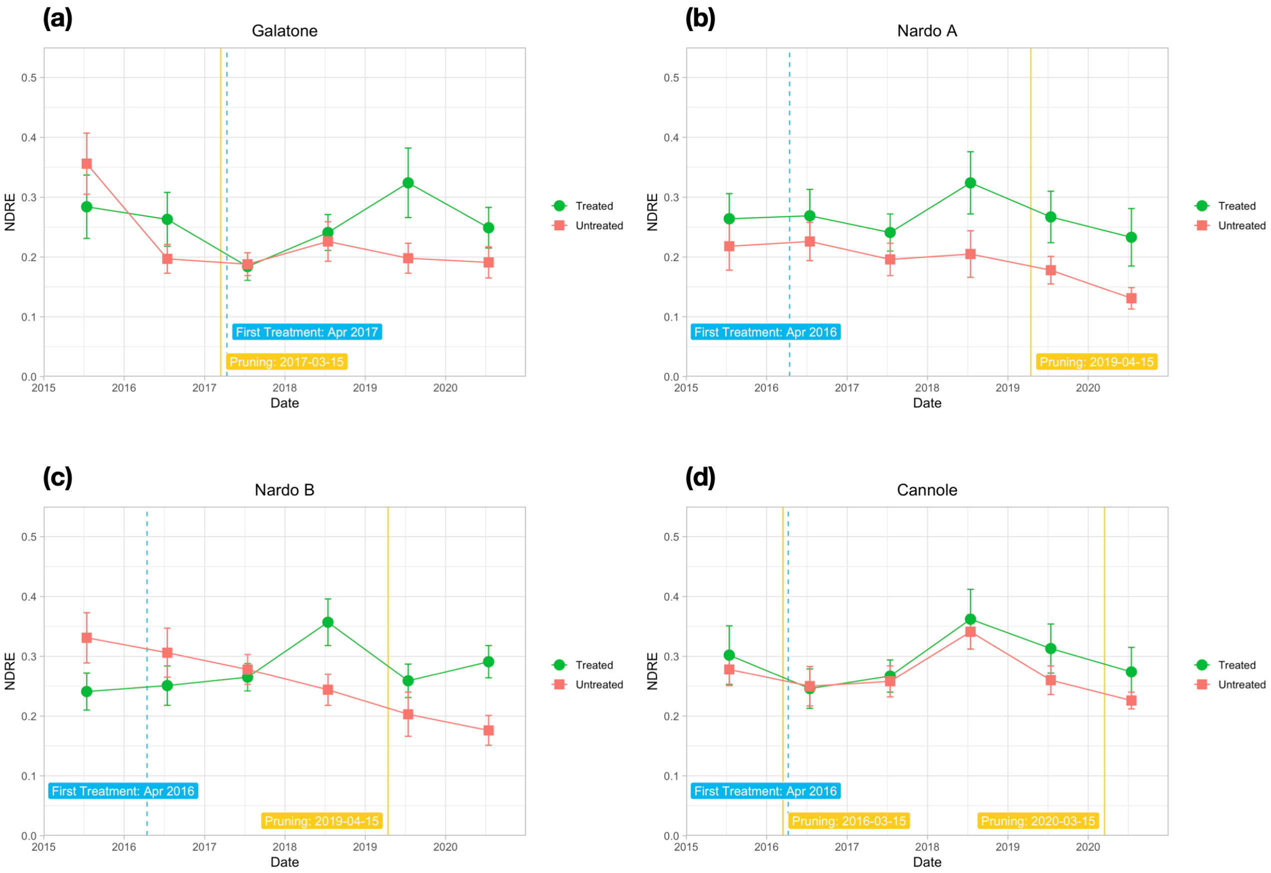

Figure S2. S-2 NDRE trend from each site in different fields with or without restoration treatment. **(a)** Galatone; **(b)** Nardò_A; **(c)** Nardò_B; **(d)** Cannole. Starting period for the restoration treatment and pruning date are indicated. Sites **(a)**, **(b)**, **(c)** on the Ionian coast and site **(d)** on the Adriatic coast.

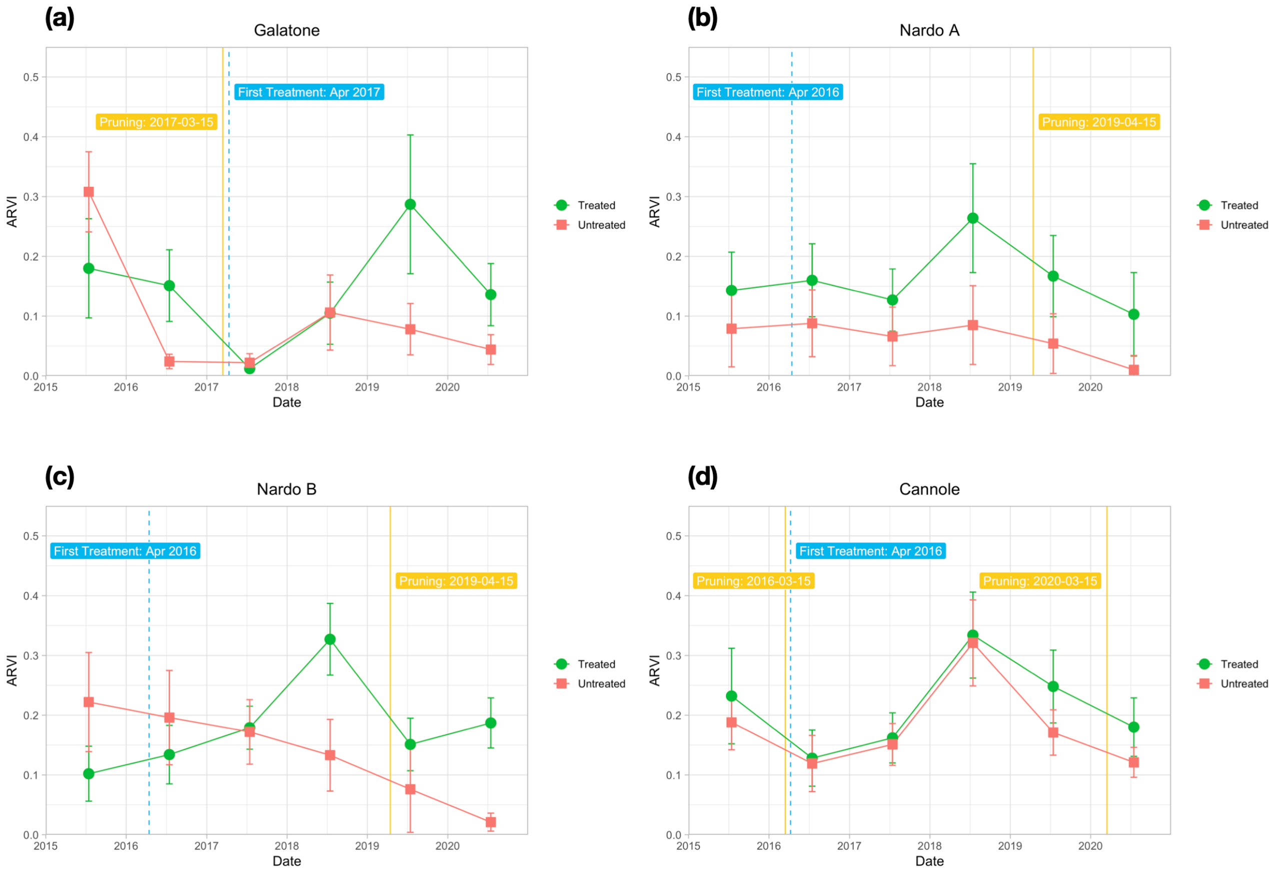

Figure S3. S-2 ARVI trend from each site in different fields with or without restoration treatment. **(a)** Galatone; **(b)** Nardò_A; **(c)** Nardò_B; **(d)** Cannole. Starting period for the restoration treatment and pruning date are indicated. Sites **(a)**, **(b)**, **(c)** on the Ionian coast and site **(d)** on the Adriatic coast.
